# Do osteohistology, microanatomy or life history traits show patterns linked to neotenic lifestyles in extant Caudata and the Permian temnospondyl *Apateon*?

**DOI:** 10.1186/s12862-026-02577-x

**Published:** 2026-10-01

**Authors:** Nicole Klein, Dorota Konietzko-Meier, Rainer R. Schoch, Eva M. Griebeler

**Affiliations:** 1 State Museum of Natural History Stuttgart, Rosenstein 1, 70191 Stuttgart, Germany; 2 Bonn Institute for Organismic Biology, Department of Palaeontology (BIOB-V), University of Bonn, Nussallee 8, 53115 Bonn, Germany; 3 Department of Palaeontology, Institute of Biology, University of Hohenheim, Garbenstrasse 30, 70599 Stuttgart, Germany; 4 Institute of Organismic and Molecular Evolution, Johannes Gutenberg- University, 55099 Mainz, Germany

**Keywords:** Petrographic ground sections, Bone matrix, Osteoblasts, Obligate neoteny, Facultative neoteny, Life history strategies, Statistical analyses

## Abstract

Neoteny is widespread in extant Caudata and has also been inferred in extinct temnospondyls. Here, we compare osteohistology, microanatomy, and life history traits of the Permian branchiosaurid *Apateon* spp. with those of modern obligate and facultative neotenic and not neotenic caudates to possibly identify common patterns separating groups. The osteohistology of these modern caudates is studied for the first time based on petrographic ground sections, which yield a better comparability between modern and fossilized bones than microtomized thin sections. We found in all adult individuals’ avascular lamellar matrix, except for *Necturus maculosus*, which grew with avascular parallel-fibered bone. The presence of osteoblasts indicates prolonged bone mineralization process in caudates. Medullary cavity size varies widely in humeri of *Ambystoma mexicanum* and in humeri and femora of *Apateon* spp., whereas in all other taxa it is more uniform and tendentially compacter which, however, might be the result of lower sample sizes. *Apateon* spp. had numerous lines of arrested growth indicating strong seasonal growth as was described in detail before and which is in accordance with the inferred environmental conditions. The markedness of growth marks is well pronounced in *Necturus maculosus*, *Amphiuma means*, *Triturus cristatus*, *Lissotriton vulgaris* and *Salamandra salamandra* but not in others. Poor expression of growth marks might be related to constant climatic conditions (e.g., *Ambystoma mexicanum*, *Proteus anguinus*) or due to an early ontogenetic stage and a yet low mineralized matrix of some individuals in our sample (*Ichthyosaura alpestris*, *Calotriton asper*). Growth mark counts of captive *A. mexicanum* of known age considerably underestimated their actual age. The analysis of adult body mass, age at maturation and maximum longevity of a large dataset on obligate neotenic and non-obligate neotenic caudates indicated a high variability in the two latter traits and associations among each other and to adult body mass. Due to this large variability, we did not find any pattern that can be used to unequivocally infer whether a taxon is obligate neotenic or not. Overall, our results suggest that no shared pattern in osteohistology, microanatomy or life history traits exists among extant obligate neotenic, non-obligate neotenic and extinct neotenic *Apateon*.

**Supplementary Information:**

The online version contains supplementary material available at https://doi.org/10.1186/s12862-026-02577-x.

## Background

Life history traits are assessable from osteohistological analyses. Besides age at maturation and age(s) at death as proxy for longevity (i.e. skeletochronological data), several other osteohistological features can be studied which include microanatomy (compact vs. porous areas; i.e., medullary cavity/region, erosional and vascular cavities) and tissue type (matrix, density and arrangement of vascular canals) (summarized in [8]). Microanatomy can inform about lifestyle (i.e., aquatic vs. terrestrial) and tissue types reveal additional data on growth, growth rates and changes along ontogeny, resulting together with -if present- growth marks, in growth patterns and strategies (summarized in [9, 11]). For fossilized bones of extinct vertebrates, these data are only assessable through petrographic ground sections. For the interpretation of osteohistological structures observed in extinct animals a comparison with living animals is essential. Living vertebrates, however, are usually studied from stained microtomized thin sections (e.g., for lissamphibians see e.g., [7, 12, 39, 49]).

Microtomized and petrographic ground sections differ in the treatment of fresh and fossilized bony samples prior to sectioning and in the final thickness of sections, both resulting in different optical features (i.e., such as visibility of mineralized collagen fibers and growth marks; [55]). In stained microtomized sections, growth marks are well visible but matrix and primary and secondary structures are more difficult to study than in petrographic ground sections. For a detailed methodological comparison of petrographic ground sections vs. stained microtomized thin sections, see Schucht et al., [55].

Age at maturation, longevity, and other growth-related parameters such as growth rates and adult body size form important life history traits of species [21]. These can be estimated from fitting growth curves to series on annual growth marks inferred from microtomized and petrographic ground sections (e.g., [9, 19, 20, 49, 58]). Life history traits are intercorrelated among each other [42], but it has been shown that associations of traits differ between different taxonomic groups (e.g., [40] and even between different reproduction modes (e.g., [20, 23]). Among the intercorrelations between traits, their relations to body mass (allometries) are best studied (e.g., [40]. In extant Lissamphibia, however, life history traits such as egg mass, clutch size, and age at maturation depend less strong on body mass than in reptiles [13, 24].

**Fig. 1:**
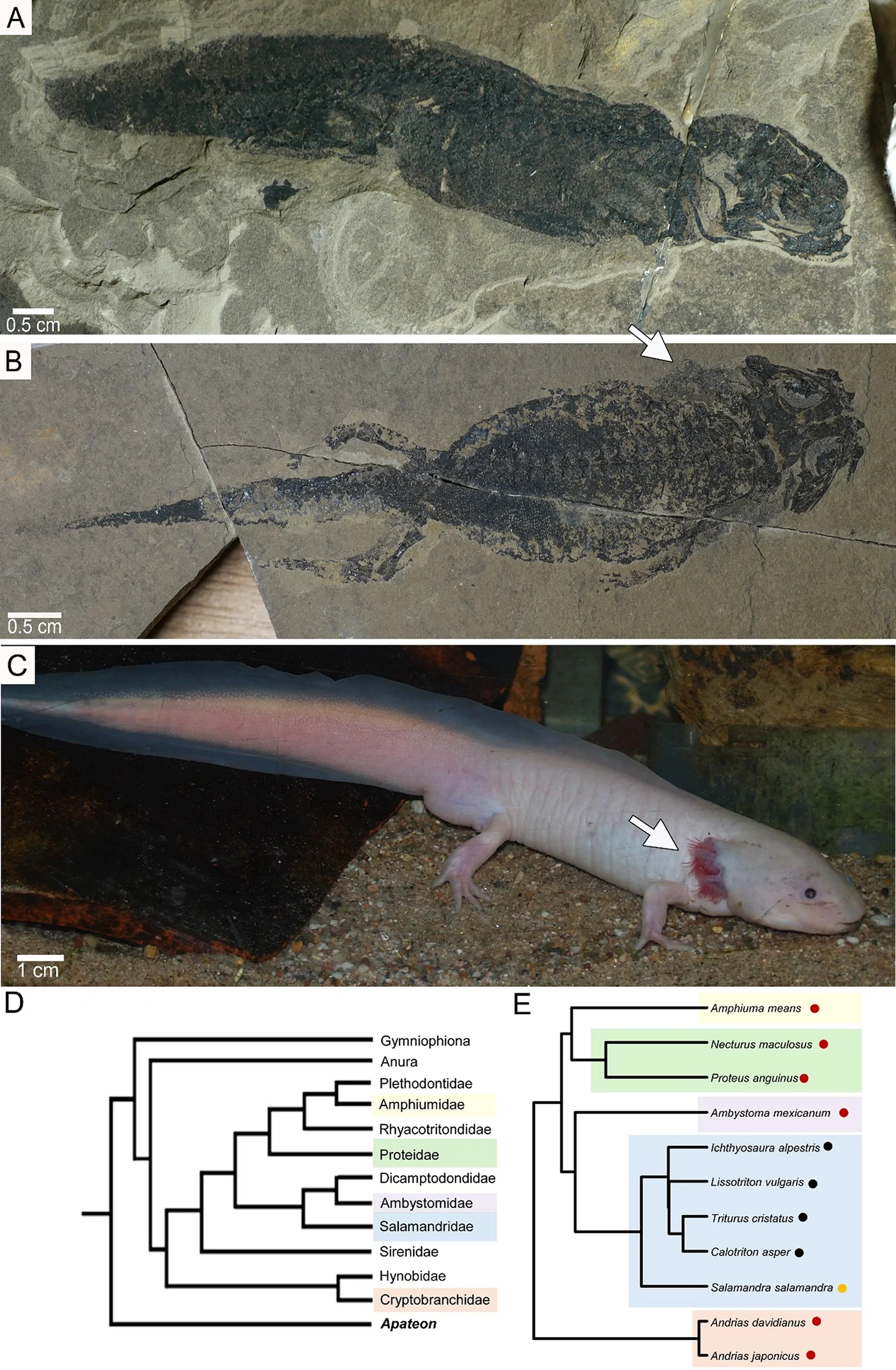
*Apateon* spp. in **A**, lateral view and **B**, dorsal view. **C**, An albino of *Ambystoma mexicanum*. Arrows point to the branchial denticle and external gills, respectively. **D**, Simplified phylogenetic relationships of Caudata. (modified from [27]) including the extinct dissorophoid *Apateon*. As a member of Dissorophoidea, *Apateon* spp. has been proposed as close to the origin of modern lissamphibians (e.g., [33]). **E**, Taxa studied in petrographic ground sections. Red point marks obligate neotenic caudates, black point marks facultative neotenic taxa, and the yellow point indicates the non-neotenic taxon. Please note that we herein consider *Ambystoma mexicanum* and *Andrias* spp. as obligate neotenic taxa following other studies (e.g., [2])

Neoteny has evolved multiple times during lissamphibian evolution [2, 34] (Fig. 1D, E). It is a form of paedomorphosis, in which somatic development is delayed compared to gonadic development and thus sexual maturation is reached while retaining larval somatic features (e.g., [22]). Neotenic caudates do not undergo a drastic and short-term metamorphosis and have a monophasic aquatic life cycle [54]. Modern neotenic caudates thus do not change their habitat from water to land but stay aquatic during their entire life. Metamorphosis, which includes biphasic or multiphasic life cycles, is considered the plesiomorphic condition in Lissamphibia ([44, 60]; but see [38]). Neoteny is thus an advanced alternative developmental pathway during which metamorphosis is suppressed due to heterochronic shifts in development [7, 52]. Among modern caudates, obligate neoteny is rare but phylogenetically widespread and found in all major groups (Fig. 1D, E). However, in many modern caudates neoteny is a facultative pathway and if this developmental mode is applied by an individual strongly depends on e.g., environmental conditions (e.g., [35, 36, 56].

The Lower Permian branchiosaurid temnospondyl *Apateon* spp. (*A. pedestris* and *A. caducus*) resembles the gestalt of modern neotenic ambystomatid *Ambystoma mexicanum* (the axolotl, Fig. 1A-C). Besides *Apateon* spp. having a smaller body size, both taxa share short limbs, strong swimming tails, but more importantly, the lifelong retention of larval characters (Fig. 1A-C; [18, 46, 52, 54]). In extinct *Apateon* spp., neoteny is well documented by ontogenetic series showing overall poor ossification of skull and postcranium and the lifelong presence of a larval hyobranchial apparatus including branchial denticles (summarized in [52]) (Fig. 1A, B). Morphological evidence of neoteny in *Apateon* spp. is further supported by histological data indicating sexual maturation in individuals showing still larval characters [46]. *Apateon* spp. are among the most ancient unequivocally documented neotenic tetrapods to date [3, 50, 54]. Metamorphosed specimens of *Apateon* spp. are not known so far but of other branchiosaurids (*Xerodromeus gracilis*; [52, 53]). This indicates obligate neoteny in *Apateon* spp., however, we cannot rule out that this taxon was facultative neotenic.

Whether neotenic taxa exhibit consistent osteohistological features and associations of life history traits are of particular interest for identifying neoteny in extinct taxa and for understanding the evolution of neoteny. As mentioned above, histological studies of lissamphibians and especially of neotenic taxa based on petrographic ground sections remain scarce [7, 31] but are essential for comparison of osteohistological features of extinct and modern taxa.

The current study focuses on the question of whether obligate neotenic caudates share common patterns in bone histology, microanatomy or life history traits and whether these separate them from facultative and non-neotenic taxa. To this end, (1) we compared the long-extinct neotenic branchiosaurid *Apateon* spp. to selected obligate and facultative neotenic extant caudates and to one species which is not neotenic. The osteohistological analyses focus on four obligate neotenic species (*Ambystoma mexicanum*, axolotl, Ambystomidae; *Proteus anguinus*, olm, Proteidae; *Necturus maculosus*, common mudpuppy, Proteidae; *Amphiuma means*, two-toed Amphiuma, Amphiumidae), three facultative neotenic newts (*Lissotriton vulgaris*, smooth newt, Salamandridae; *Triturus cristatus*, northern crested newt, Salamandridae; *Calotriton asper*, Pyrenean brook newt, Salamandridae; *Ichthyosaura alpestris*, Alpine newt, Salamandridae) and the viviparous *Salamandra salamandra* (fire salamander, Salamandridae) as non-neotenic taxon (Tables 1, 2 and 3). Osteohistological characters of these modern caudates are studied for the first time in petrographic ground sections. For potential differences in life history traits and strategies between obligate neotenic caudates and caudates that are not obligate neotenic, (2) we analyzed a large dataset on adult body masses, maximum longevities and ages at maturation of modern caudates. Specifically, we explored differences in the allometric scaling of maximum longevity and age at maturation to adult body mass and whether both groups show specific associations in species’ maximum longevities and ages at maturation that separate them from each other. As neoteny evolved multiple times in the evolutionary history of modern caudates ([2, 34], Fig. 1D, E), our study is based on the hypothesis that functional demands such as comparable lifestyles and similar developmental pathways might have led to convergent osteohistological features and/or life history traits in obligate neotenic caudates.

## Materials and methods

### Osteohistological analysis

The samples of the branchiosaurid temnospondyl *Apateon* spp. (*A. pedestris* and *A. caducus*) originated from sediments from the Lower Permian Saar-Nahe Basin (localities Niederkirchen M6-N, Erdesbach M7-E, and Odernheim M8-OKB). For more information on the environment and the stratigraphical sequence of the respective localities, see Schoch, [51]. The petrographic thin sections were studied and published before by Sanchez et al., ([46–48]) and age at maturation and age at death for *Apateon* spp. were taken from these studies (Table 1).

**Table 1:** Environments used and life history data of *Apateon* spp. and extant Caudata studied

	*Apateon* spp.	*Ambystoma mexicanum*	*Necturus maculosus*	*Prote* *us* *anguinus*	*Amphiuma means*	*Calotriton asper*	*Ichth* *yosaura* *alpestris*	*Liss* *otriton* *vulgaris*	*Tritu* *rus* *cristatus*	*Salama* *ndra* *salamandra*
habitat, environment, geographic distribution	lakes at high altitude (2000 m); poorly oxygenated. today region of Saar-Nahe Basin, Germany, France, Europe; during Lower Permian tropical region	cool, well oxygenated, flat lakes with low currents at about 2240mNN; endemic to Xochimilco-and Chalco-lake, remains of a Vulcanic basin close to Mexico City, Mexico	well oxygenated clear and cold-water bodies with stony or muddy ground; East of N/mid USA and SE Canada	underground water systems in karst formations; endemic to Dinaric Alps, SE Europe	near coastal swamps and pools; S/SE of USA.	cold, oxygen-rich, mountain streams and lakes. mountain ranges of the Pyrenees	prefers forests; needs water bodies for reproduction; low and highlands; Central Europe	prefers forests; needs water bodies for reproduction; low and highlands; Europe and neighboring countries	prefers forests; needs water bodies for reproduction; low and highlands; Central Europe	prefers forests; needs water bodies for reproduction; low and highlands; south and Central Europe
**temperature** **range**	highly seasonal with cold winters	14°-16°/12°-22° sensitive against temp. fluctuations; slightly seasonal	activity range: 9°-20 °C. hibernates on the ground of water bodies at 0–4 °C. intolerant at higher temperatures: hides/aestivates	between 8 °C (winter) and 11 °C (in one locality rarely up to 14 °C in summer); lives under fairly constant temperatures	prefers 23.5°-28.3 °C; reduces activity below 15 °C but not totally inactive	does not tolerate temperaturesabove 16 °C; hibernates	temperate belt of Europe; hibernates	temperate belt of Europe and neighboring countries; hibernates	temperate belt of Europe; hibernates	temperate belt of south and central Europe; hibernates
**lifestyle**	aquatic; ?ground dwellers	aquatic; ground dweller	aquatic; active predator	aquatic; ground dweller; very inactive animals	aquatic; active predator	aquatic; active predator	terrestrial, aquatic during reproduction	terrestrial, aquatic during reproduction	terrestrial, aquatic during reproduction	terrestrial, partially aquatic during reproduction
**body size range** **body mass**	80–150 mm; 3–10 g	230–280 mm; 60 g	300 mm; some > 500 mm; 162 g	230 –205 mm; max 300 mm 15 g	> 1 m 480 g	males 105–120 mm; females 110–140 mm; 7 g	70–120 mm; 1–4 g	80–110 mm; 2–3 g	males 135 mm; females 160 mm; 4–19 g	150–250 mm; females larger than males; 40 g
**paedomorphosis**	facultative/ obligate neotenic	obligate neotenic (can metamorphize but usually does not in nature)	obligate neotenic	obligate neotenic	facultative neotenic	facultative neotenic in some mountain lakes but not in streams	facultative neotenic	neoteny is not documented	facultative neotenic	no successful breeding in neotenics documented
**clutch size**	unknown	> 550 eggs/p.a.	+ 35/120 eggs average 60–93/ p.a.; sometimes biannual	35 eggs every 12 yrs	~ 200 eggs/ p.a.	20 to 30 eggs/p.a.	73–190 eggs/ p.a.	60–300 eggs/ p.a.	70–600 (average 150–200) eggs/p.a.	10–70 larvae (average 20–40); up to 20 metamorphs/p.a.
**mode of reproduction** **hatchling size** **hatching time**	unknown; 10.3 mm Cejp (pers. com.)	oviparous; 60–90 mm; hatch after 14–21 days at 16–18 °C	oviparous; ~ 20 mm; hatch after 1–2 month	oviparous; larval size and egg development unknown	oviparous; ~ 50 mm; hatch after 5–6 month, females breed	oviparous; 3.5–5 mm; hatching time and size depends on elevation	oviparous; 7–11 mm; hatch after 2–4 weeks	oviparous; 5–7 mm; hatch after 10–20 days	oviparous; 8–10 mm; hatch after 2–3 month	ovoviparous and viviparous; larvae 20–30 mm and metamorphs 40–70 mm
**metamorphosis**	no	usually not (but can be induced)	no	no	no	depends on elevation 1.5 year to 3 yr	usually yes 1st or 2nd yr	usually yes 1st or 2nd yr	usually yes 1st or 2nd yr	usually yes 1st yr or born as metamorphs
**sexual maturity**	depending on locality between 5–7 yrs; (Sanchez et al., [46, 47])	8–15 month	6–8 yrs	~in average at 15.6 yrs	at 3–4 yrs	2 yrs at 1000 m, male 2.5-3 yrs; females 4 yrs at 2000 m	at 2–3 yrs	at 3 yrs	at 2–3 yrs	at 5–6 yrs
**longevity**	up to 9 yrs according to Sanchez et al., ([46, 47)	over 10 yrs; 19 + yrs (still alive) NK (pers. obs.)	~ 20/34yrs	average 68.5 yrs; 100 yrs recorded	13–27 yrs	~ 20/35 yrs	9–11 yrs	14 yrs	7–9/17 yrs	24/50 yrs

The information for the temnospondyl branchiosaurid *Apateon* spp. (*A. pedestris* and *A. caducus*) are based on Boy and Sues, [5], Boy, [4], and Sanchez et al., ([46, 47]). The information for the modern Caudates included here are from the homepage amphibiaweb.org [1], if not cited otherwise, see also Supplementary information SI. Average body masses are taken from Cejp and Griebeler, [13]. Average body mass for *Apateon *spp. was downscaled from *Ambystoma mexicanum*.

Five individuals (four males, larva of unknown sex) of *Ambystoma mexicanum* (BIOP-PAL sample, ZFMK 83269), three individuals of *Proteus anguinus* (ZFMK 86031/unknown sex, ZFMK 86071/unknown sex, ZFMK 86001/female), and three individuals of *Triturus cristatus* (BIOP-PAL-182a-c) representing different ontogenetic stages were sampled. A male (ZFMK 83280) and a female (ZFMK 83281) of *Necturus maculosus*, one individual of *Amphiuma means* (ZFMK 32181/unknown sex), one individual of *Calotriton asper* (ZFMK 39157/unknown sex), one individual of *Ichthyosaura alpestris* (BIOP-PAL-180), and one individual of *Salamandra salamandra* (BIOP-PAL-178) were studied as well. Bones of modern Caudata were kindly provided from the collection of the ZFMK and from the BIOP-V. The *Ambystoma mexicanum* sample now housed at the BIOP-V, were individuals bred and kept for their entire life by one of the authors (NK) (Table 2). These axolotls experienced room temperatures ranging from ~ 15–25 °C throughout the year and all lived lifelong together in the same tank. They all died due to natural reasons (old age, or at least no obvious other reasons) and were immediately frozen after death. The specimens of *Ichthyosaura alpestris* and *Salamandra salamandra* were found dead in nature and were collected by one of us (NK; Eastern Vulcanic Eifel, Central Germany). They were frozen until prepared for thin sectioning. The specimens of *Triturus cristatus* were old lecture specimens glued as an ontogenetic series to a paper wall. They dried over the decades, resulting in poor preservation. *Lissotriton vulgaris* is the only modern taxon included for which no petrographic thin sections are available, and which was not studied firsthand but osteohistological data were inferred from Hagstrôm [25]. The ZFMK individuals originated from captive housing but were originally taken from the wild according to the records of the ZFMK, except for the *A. mexicanum* specimen which comes from a laboratory (rearing conditions are unknown) and *Calotriton asper* which was collected in the Pyrenees. The ZFMK specimens had been stored in ethanol for decades (most of them since the mid-70ties), which might result in an underestimation of their body mass and body size (Table 2) due to shrinking.

The specimens of the facultative neotenic newts are all metamorphized individuals. Please note that we consider *A. mexicanum* as obligate neotenic, although we are aware that it can be forced by a special treatment to metamorphize. *Andrias* spp. is in our study also treated as an obligate neotenic taxon despite the resorption of its external gills during ontogeny (following [2]).

Life history data including ages at maturation and longevities of modern samples were taken from literature (see Table 1; Supplementary information SI). Although some of the modern samples show growth marks in the petrographic ground sections, we do not further analyze the preserved growth record in this study. The growth record for *Apateon* was analyzed before by Sanchez et al., [46, 47] and the data on age at maturation and age at death given in Table 1 for *Apateon* spp. are adopted from these studies.

All studied bones of modern taxa had been micro-Ct scanned prior to thin sectioning at the BIOP-V to identify the best sampling location along the shaft. The elements were dissected personally by us from the respective specimens, defleshed mechanically without any chemical treatment and were not decalcified. After having been defleshed, elements were dried in a heating cabinet for 48 h at 50 °C. All bones were then proceeded into petrographic ground sections at the BIOP-V following petrographic thin-sectioning techniques as described in Klein and Sander [30]. For details on taxon-specific life history traits of the here included taxa, see Table 1 and the Supplementary information (SI). Table 2 summarizes details on body size and mass, age at death if known, sampled element and life conditions of the respective modern samples.

The percentage value of the size of the medullary cavity was calculated based on its measured diameter length in relation to the longest diameter of each cross section (usually the lateromedial diameter). For estimating the body mass of *Apateon* spp., the body mass of specimen ZFMK 83269 (*Ambystoma mexicanum*; Table 2) was downscaled thereby assuming a similar body shape in both taxa.

All specimens (except for *Lissotriton vulgaris*, see above) were analyzed using standard osteohistological methods for petrographic thin sections as described in detail in Francillon-Vieillot et al., [16] and Buffrénil and Quilhac, [8]. The osteohistological analysis was performed with a Leica DM LP and a Leica DMP 750 polarizing light microscope. The pictures were obtained with a Leica microscope DM LP equipped with an EOS Canon 2000D camera. The histological nomenclature follows Francillon-Vieillot et al., [16] and Buffrénil et al., [6].

**Fig. 2:**
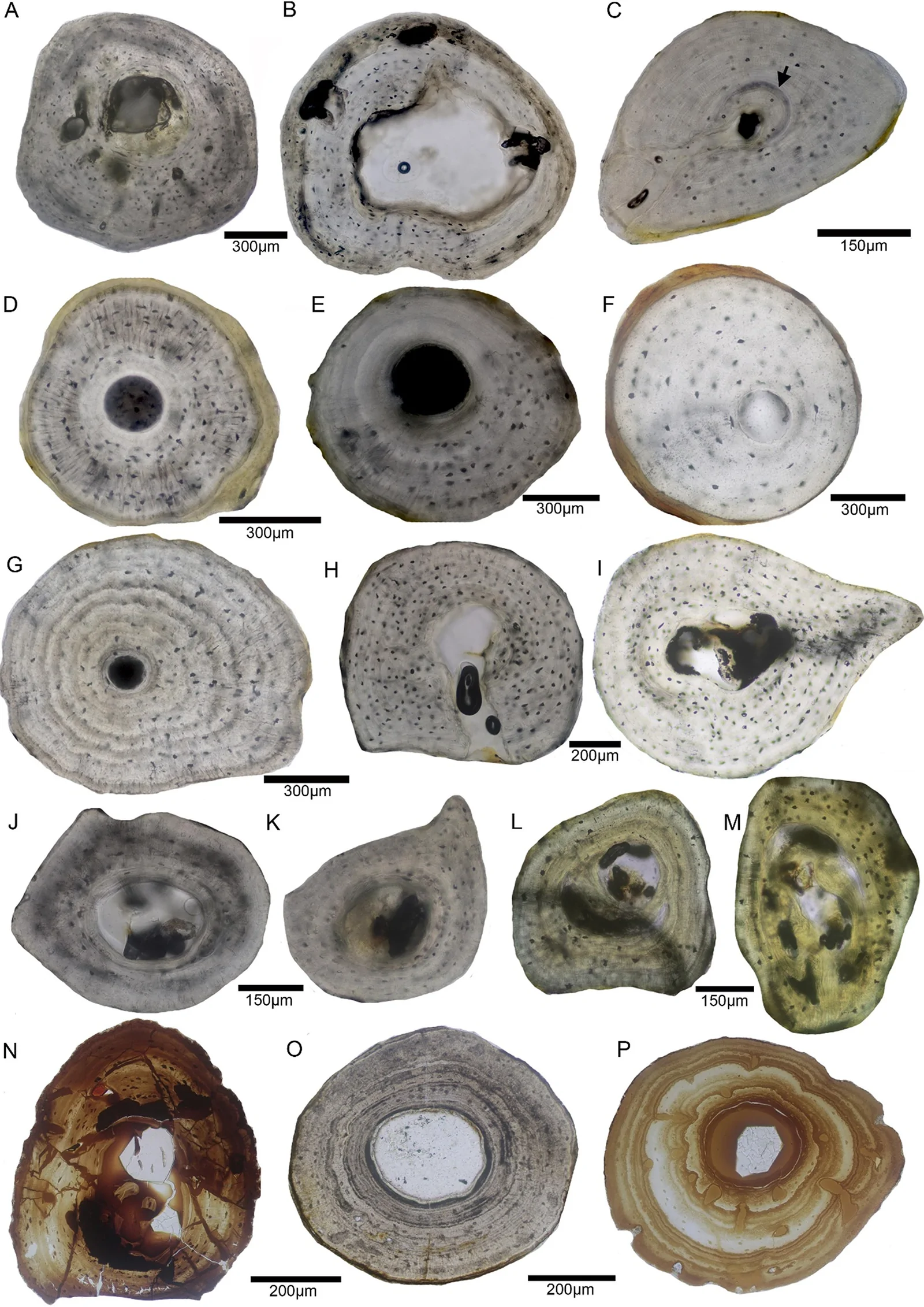
Petrographic ground sections of midshaft areas of selected taxa. All sections are displayed in plane polarized light. Medullary cavities are in all samples located rather centrally. The black “points” scattered throughout the cortices are osteocyte lacunae. **A**, Humeral cross section of *Ambystoma mexicanum ***(**BIOP-PAL-176). **B**, Humeral cross section of *Ambystoma mexicanum ***(**BIOP-PAL-175). Note the difference in the size of the medullary cavities. **C**, Humeral cross section of *Calotriton asper* (ZFMK 39157). The arrow marks the metamorphosis line. **D**, Humeral cross section of *Proteus anguinus* (ZFMK 86071). **E**, Humeral cross section of *Proteus anguinus* (ZFMK 86001). **F**, Femoral cross section of *Amphiuma means* (ZFMK 32181). **G**, Humeral cross sections of female *Necturus maculosus* (ZFMK 83281). **H**, Humeral cross section of *Salamandra salamandra* (BIOP-PAL-178). **I**, Femoral cross section of *Salamandra salamandra* (BIOP-PAL-178). **J**, Humeral cross section of *Ichthyosaura alpestris* (BIOP-PAL-180). **K**, Femoral cross section of *Ichthyosaura alpestris* (BIOP-PAL-180). **L**, Humeral cross section of *Triturus cristatus* (BIOP-PAL-182a). **M**, Femoral cross section of *Triturus cristatus* (BIOP-PAL-182a). **N**, *Apateon pedestris* (SMNS 54981) from Odernheim. **O**, *Apateon pedestris* (SMNS 55018) from Niederkirchen. **P**, *Apateon caducus* (GPIM 1572) from Erdesbach

Following that, the identification of the bony matrices in petrographic thin sections, relies exclusively on the orientation of their mineralized collagenous fibers, which are best visible in polarized light microscopy. Collagenous fibers in lamellar matrix are highly organized and show in polarized light an alternating birefringence (dark-bright pattern) (Fig. 2A-C), whereas parallel-fibred matrix is less highly organized and shows wider areas of the same birefringence (Fig. 2D). Lamellar matrix is always highly organized but parallel-fibred matrix can vary between low organized to very highly organized then grading into lamellar matrix. This sometimes makes the exact identification of this matrix type difficult.

**Table 2:** List of petrographic ground sections of humeri and femora taken at midshaft region of studied modern Caudata

Specimens	body size and mass	age/gmc	element/ csdmc	comments
***Ambystoma mexicanum***				
BIOP-PAL-186 (undetermined sex)	55 mm ~ 1.5 g	3 months/ 0 gmc	humerus/ 60%	captive conditions controlled
ZFMK 83269 (male)	180 mm 33 g	unknown/ 4 changes in color	humerus/ 80%	captive conditions unknown
BIOP-PAL-175 (male)	230 mm 160 g	14 years/ 7-8 gmc	humerus and femur/ 60% & 65%	captive conditions controlled
BIOP-PAL-176 (male)	250 mm 224 g	16 years/ ~ 10 gmc	humerus and femur/ 30% & 65%	captive conditions controlled
BIOP-PAL-177 (male)	230 mm 189 g	18 years/ not possible to count	humerus and femur/ 80% & 65%	captive conditions controlled
***Necturus maculosus***				
ZFMK 83280 male	255 mm 99 g	unknown age/ 8-9 gmc	humerus/ 36%	unknown conditions; wild-caught, died in captivity
ZFMK 83281 female	250 mm 82 g	unknown age/ 7-8 gmc	humerus/ 12%	unknown conditions; wild-caught, died in captivity
***Proteus anguinus***				
ZFMK 86031 unkown sex	180 mm 11 g	unknown age/ no gmc	humerus/ 25%	unknown conditions; wild-caught, died in captivity
ZFMK 86071 unkown sex	230 mm 8 g	unknown age/ 5-6 gmc	humerus/ 25%	unknown conditions; wild-caught, died in captivity
ZFMK 86001 female	270 mm 47 g	unknown age/ 7-8 gmc	humerus/ 23.5%	unknown conditions; wild-caught, died in captivity
***Amphiuma means***				
ZFMK 32181 unknown sex	580 mm 173 g	unknown age/ 5-6 gmc	femur/ 14%	unknown conditions; wild-caught, died in captivity
***Calotriton asper***				
ZFMK 39157 unknown sex	130 mm 7 g	unknown age 5 gmc	humerus/ 10%	wild-caught in Pyrenees
***Triturus cristatus***				unknown conditions
BIOP-PAL-182a adult	90 mm na	unknown age/ 3 gmc	humerus and femur 35.8% & 58.4%	unknown conditions
BIOP-PAL-182b not fully grown	70 mm na	unknown age/ no gmc	femur 65%	unknown conditions
BIOP-PAL-182c not fully grown	43 mm na	unknown age/ no gmc	humerus and femur 41.6% & 64%	unknown conditions
***Ichthyosaura alpestris***				
BIOP-PAL-180	90 mm 12 g	unknown age/ 2–3 gmc	humerus and femur 40 & 37.5%	wild, found dead unknown conditions
***Salamandra salamandra***				
BIOP-PAL-178	160 mm 45 g	unknown age/ 6–7 gmc	humerus and femur 26.6% & 38.7%	wild, found dead unknown conditions

Abbreviations: csdmc, cross sectional diameter of the medullary cavity; gmc, growth mark count; na, not available

### Statistical analysis on life history traits

We conducted several statistical analyses to find out whether life history traits and their associations found in species differ between extant obligate neotenic Caudata and Caudata that are not obligate neotenic (all other species, hereafter non-obligate neotenic species). We therefore compiled a dataset on species’ adult body mass (in grams), maximum longevity (in years), age at maturation (in years) and development mode (obligate neotenic vs. non-obligate neotenic).

Our dataset combines species’ adult body masses, maximum longevities and ages at maturation from the datasets of Cejp and Griebeler, [13, 14] and information on species’ developmental modes from the dataset of Liedtke et al., [34]. Authors of these studies have compiled their large datasets from the literature. Adult body masses, maximum longevities and ages at maturation of each species are average values that Cejp and Griebeler [13, 14] had calculated across several studies. The obligate neotenic caudates in our dataset lack a post-metamorphic adult phase and their reproduction mode is aquatic, whereas the non-obligate neotenic caudates comprise species for which these authors scored their reproduction mode as aquatic, semi-terrestrial, terrestrial, direct developing, or live bearing. Facultative neotenic species were assigned to the non-obligate neotenic species sample, as their life history traits are likely average values across neotenic and metamorphosing individuals.

The dataset analyzed here includes representatives of nine Caudata families and consists of 141 (16 obligate neotenic, 125 non-obligate neotenic) species for which we had their adult body mass, maximum longevity and development mode and of 161 (16 obligate neotenic, 146 non-obligate neotenic) species for which we had their adult body mass, age at maturation and developmental mode. Particularly, adult body masses, maximum longevities, ages at maturation and developmental modes of all nine modern taxa subject to our osteological examination are in our dataset. To assess adult body size, we preferred adult body mass over measures on body length (SVL) as caudates can substantially differ in their body shapes (e.g., the snake-like *Proteus anguinus* vs. the plump *Ambystoma mexicanum*) and as Cejp and Griebeler [13, 14] have estimated masses of many species for which they had found SVL in the literature only by applying the allometries on adult body mass against SVL of [61].

Prior to all statistical analysis done in R version 4.5.3 (R Core team 2026), species’ life history data were log10-log10-transformed. In all phylogeny-informed statistics, we applied the large-scale phylogenetic tree on modern lissamphibians published by Jetz and Pyron [29]. We used the Integrated Taxonomic Information System website (itis. gov) to identify synonymous species names and adopted the nomenclature of this phylogenetic tree for our final dataset.

We first calculated phylogeny-informed allometric models (PGLS models, following [43]) for three samples on Caudata and two life history traits taken from our dataset. Species samples were all caudates, all caudates except for the obligate neotenic species (non-obligate neotenic species) and the obligate neotenic species only, and the two life history traits were maximum longevity and age at maturation. We applied adult body mass as (continuous) predictor in a linear model for each sample and for both traits. For phylogenetic correction of models, we pruned the lissamphibian phylogenetic tree to the species in the respective sample. We then applied Pagel’s *λ* [62] to convert this phylogeny into a correlation structure by using the function *corPagel* (*ape*-package; [37]). Allometries inferred are linear phylogenetic generalized least squares (PGLS) models and were calculated by applying the function *glm* (*nlme*-package, [41]). We calculated 95% confidence intervals of estimated slopes, intercepts and *λ* values for all models by applying the function *intervals*. For testing whether statistically non-zero slopes and intercepts differ between respective models calculated for obligate neotenic and non-obligate neotenic caudates, we compared their 95% confidence intervals on slopes and intercepts.

To test for differences in mean values of maximum longevity and age at maturation between obligate neotenic and non-obligate neotenic caudates after accounting for an allometric effect of adult body mass on the respective trait, we conducted phylogeny-informed analysis of covariance (pANCOVA) following Revell and Harmon [43]. To this end, we calculated a PGLS model for all caudates and each trait as described above, except for using adult body mass as continuous predictor and obligate neotenic vs. non-obligate neotenic as discrete predictor of the trait. The two PGLS models inferred were then evaluated with the *anova* function for differences in means of mass-corrected trait values between obligate neotenic and non-obligate neotenic caudates. To assess differences in the dispersion of residuals between obligate neotenic and non-obligate neotenic caudates from the allometry on all caudates for both traits, we calculated for each species in the respective sample the difference between its trait value and the value predicted by the PGLS model on all caudates for the trait from its mass (residual value). We then conducted Ansari-Bradley tests (function *ansari.test*) on variance homogeneity of residuals across species samples for both traits, as residuals were not normally distributed (function *shapiro.test*) for both species’ samples and each of the traits.

Finally, we checked whether life history strategies of obligate neotenic and non-obligate neotenic species differ by running a Wilks’ λ test to which we passed each species’ residual value on maximum longevity and age at maturation. For conducting this test, we applied the function *wilks.test* (rrcov-package, [57]).

## Results

### Osteohistological analysis

#### The Permian branchiosaurid temnospondyl *Apateon* spp.

Sanchez et al. [46, 47] described avascular lamellar bone for *Apateon pedestris* and *A. caducus* (Fig. 2N-P). Numerous growth marks, as documented in their humeri and femora, indicate a strong seasonal dependency of growth in *Apateon* spp. (Table 1; Supplementary information SI). The sizes of the medullary cavities of humeri of *A. caducus* range from 17 to 33% (*n* = 3; GPIM 1297, GPIM-N 1234, GPIM 1572) for Erdesbach samples, and the only humerus measured from Niederkirchen (SMNS 55018) has a size of 40%. One femur of *A. pedestris* from Erdesbach (GPIM-N 1618) has a size of 35% and one femur from Odernheim (SMNS 54981) a size of 55%.

#### Modern Caudata

We here give only a short synthesis of the observed osteohistological and microanatomical features of the modern caudates studied. A detailed osteohistological description is included in the Supplementary information SII and a summary in Table 3. Petrographic ground sections of selected specimens are shown in Fig. 2, however, all specimens studied are figured in the Suppl. Fig. S1-3.

**Table 3:** Summary of osteohistological characters of studied taxa

	*Apateon* spp.	*Ambystoma* *mexicanum*	*Necturus maculosus*	*Prot* *eus* *anguinus*	*A* *mphiuma means*	*Calotriton* *asper*	*Lissotriton vulgaris*	*Triturus cristatus*	*Ichthyosaura alpestris*	*Salamandra* *salamandra*
matrix	lamellar matrix	cartilage in larva; varying degree of mineralization of lamellar matrix in adult individuals	parallel-fibred matrix	lamellar matrix	lamellar matrix	lamellar matrix	lamellar matrix	lamellar matrix	lamellar matrix	lamellar matrix
vascularity and erosion	avascular, except for a few specimens that show some primary vascular cavities and erosion	avascular, except for some erosion cavities	avascular	avascular	avascular	avascular	avascular	avascular	avascular	avascular
size of medullary cavity	*A. caducus* humeri: 17–33%; *A. pedestris* humeri:35–40% femur: 55%	humeri: 30–80%; femora: about 65%	male humerus: 36%; female humerus: 12%	humerus: 23.5–25%; femur not sampled	humerus: 14%; femur not sampled	humerus 10%	humerus 45%	humeri 35.8–41.6% femora 58.6, 65%, 64%	humerus 40% femur 37.5%	humerus 26% femur 38.7%
growth marks	pronounced numerous growth marks; depending on locality alternating annuli and zones or LAGs, additional rest lines in annuli; indicating strong seasonal growth	alternating zones and annuli; not very pronounced; growth mark count does not meet actual age	alternating zones and annuli ending in distinct LAGs	alternating zones and annuli/LAGs	alternating zones and annuli	alternating zones and annuli, not very pronounced in high organized lamellar matrix; numerous distinct growth marks in stained microtomized sections	distinct LAGs (in stained microtomized section)	alternating zones and annuli ending in LAGs, well visible	alternating zones and annuli; not very pronounced	alternating zones and annuli ending in LAGs, well visible

Data for *Apateon* spp. were partially taken from Sanchez et al., [46, 47] and are based on own observations. The size of the medullary cavities in *Apateon* spp. was measured for this study (see result section). Osteohistological data for modern caudates were solely obtained herein. Please note that lissamphibians are well known for their developmental plasticity and that we study here only a single or few individuals of each taxon. It is unlikely that our observations reflect the entire spectrum of osteohistological and microanatomical features possible for each taxon

### Size of medullary cavity

The size of the medullary cavities is used as a proxy for microanatomy in the otherwise avascular samples. The smaller the size of the medullary cavity is, the more compact is the bone. It is smallest in the humerus of *Calotriton asper* (10%) (Fig. 2C), followed by the humerus of the female of *Necturus maculosus* (12%) (Fig. 2G), and by the humerus of *Amphiuma means* (14%) (Fig. 2F). The size of the medullary cavities is moderate in the humeral sections of the three individuals of *Proteus anguinus* (23.5–25%) (Fig. 2D, E), in the humerus of the male of *Necturus maculosus* (36%), in the humerus of *Lissotriton vulgaris* (45%), in the humerus (40%) and femur (37.5%) of *Ichthyosaura alpestris* (Fig. 2J, K) and in the humerus (26.6%) and femur (38.7%) of *Salamandra salamandra* (Fig. 2H, I). The size of the medullary cavities in the five individuals of *Ambystoma mexicanum* varies from moderate to large in the humeri (30–80%) (Fig. 2A, B) and is rather constantly large in the femora (~ 65%) (Suppl. Fig. S1). The size of the medullary cavities in the three individuals of *Triturus cristatus* is larger in the femora (58.4%, 65%, 64%) than in the humeri where it is moderately sized (35.8%, 41.6%) (Fig. 2L, M; Suppl. Fig. S3A-F).

### Matrix

**Fig. 3:**
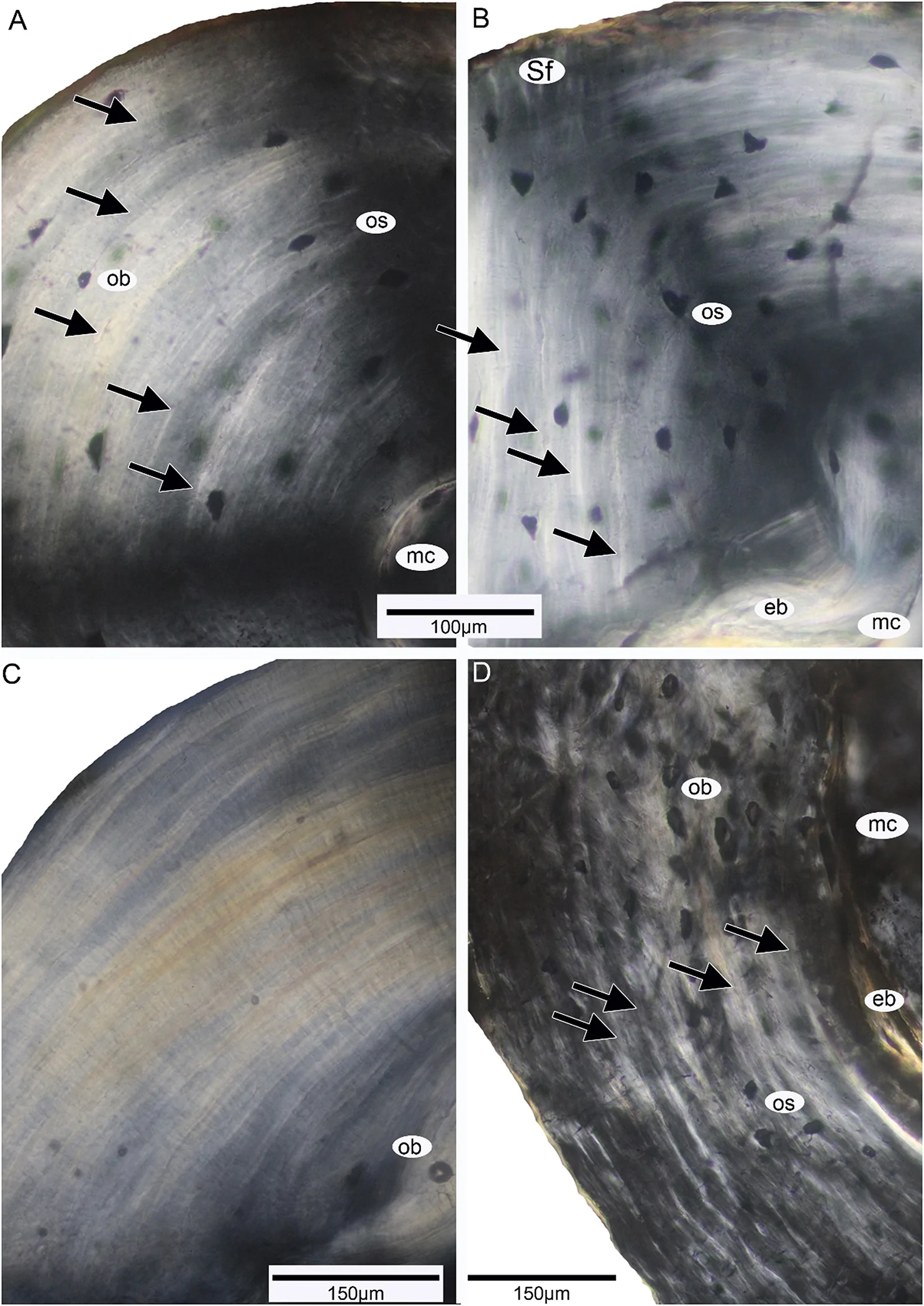
Details of matrix in petrographic ground sections of modern caudates. All figures are in cross polarized light. Arrows marks growth marks. **A**, Detail of avascular lamellar matrix in the femur of *Amphiuma means* (ZFMK 32181). **B**, Detail of avascular lamellar matrix in the humerus of *Salamandra salamandra* (BIOP-PAL-178). **C**, Detail of avascular lamellar matrix in the humerus of *Calotriton asper* (ZFMK 39157). Note the extremely high organization of this lamellar bone well visible in comparisons to A and B. **D**, Detail of parallel-fibred matrix in the humerus of female of *Necturus maculosus* (ZFMK 83281). Please note the differences in grade of organization between the lamellar matrices (A-C) and the parallel-fibred matrix (D). The black points represent osteocyte lacunae and transforming osteoblasts. **Abbreviations**: eb, endosteal bone; mc, medullary cavity; ob, osteoblast, os, osteocyte; Sf, Sharpey’s fibers

The type of matrix is avascular lamellar matrix (Figs. 2 and 3A-C) in all modern samples, except for *Necturus maculosus. Necturus maculosus* grew with avascular parallel-fibred bone (Fig. 3D), indicating a slightly faster deposition of matrix and thus of a higher growth rate. *Calotriton asper* has the highest organized matrix across our sample (Fig. 2C, 3C) despite showing osteocyte lacunae and transitional forms of osteoblasts developing into osteocytes within its matrix [17]. The adult individuals of *Ambystoma mexicanum* show also principally avascular lamellar bone but in different degrees of organization and mineralization (Suppl. SII; Suppl. Fig. S1). The cortical matrix is cartilaginous in the larva of *A. mexicanum* (BIOP-PAL-186), which is in accordance with the observations of Riquelme-Guzmán et al. [45]. The axolotl specimen ZFMK 83269 documents ongoing mineralization of the bone matrix as indicated by transforming osteoblasts located in the cortex. In the fully grown and old aged specimens of *A. mexicanum* (BIOP-PAL-177; BIOP-PAL-175), ongoing mineralization can be observed as well, whereas BIOP-PAL-176 has a well mineralized matrix depicting only osteocyte lacunae and no transforming osteoblasts (Supplementary information SII; Suppl. Fig. S1). The sections of the smallest specimen of *Proteus anguinus* (ZFMK 86031; Suppl. Fig. S2C, D) and of the two not fully grown specimens of *Triturus cristatus* (BIOP-PAL-182b, c; Suppl. Fig. Fig. S3C-F) consist solely of cartilage. Only the samples of *Ambystoma mexicanum* display distinct erosion (Fig. 1A; Suppl. Fig. S1).

### Growth marks

*Apateon* spp. (see also [46, 47]), *Salamandra salamandra*, and *Triturus cristatus* show distinct lines of arrested growth (LAGs) in their petrographic thin sections. The section of *Amphiuma means* and that of the female of *Necturus maculosus* both show distinct growth marks in form of zones and annuli (sometimes even LAGs) whereas growth marks in the male section of *Necturus maculosus* are only faintly expressed. In all other taxa, a stratification of the cortex, resembling alternating zones and annuli [16] are visible but not very pronounced. Identification of annual growth marks is further hampered by the high organization of the lamellar matrix, which also results in stratification of the cortex (Fig. 3C). The growth cycles found in *Ambystoma mexicanum* do not reflect the actual age of these individuals (Tables 2 and 3; Suppl. SII). *Calotriton asper* shows a change in matrix in the innermost cortex being surrounded by a distinct rest line in the petrographic section, which likely represents a metamorphosis line (Fig. 2C; [12]). For *Lissotriton vulgaris* no petrographic thin sections are available, the published photo of a stained microtomized thin section of a humerus shows well developed LAGs [25]. For more detailed information on all taxa, see Tables 2 and 3 and the Supplementary information SII.

### Differences in life history traits and strategies between extant obligate neotenic and non-obligate neotenic caudates

**Fig. 4:**
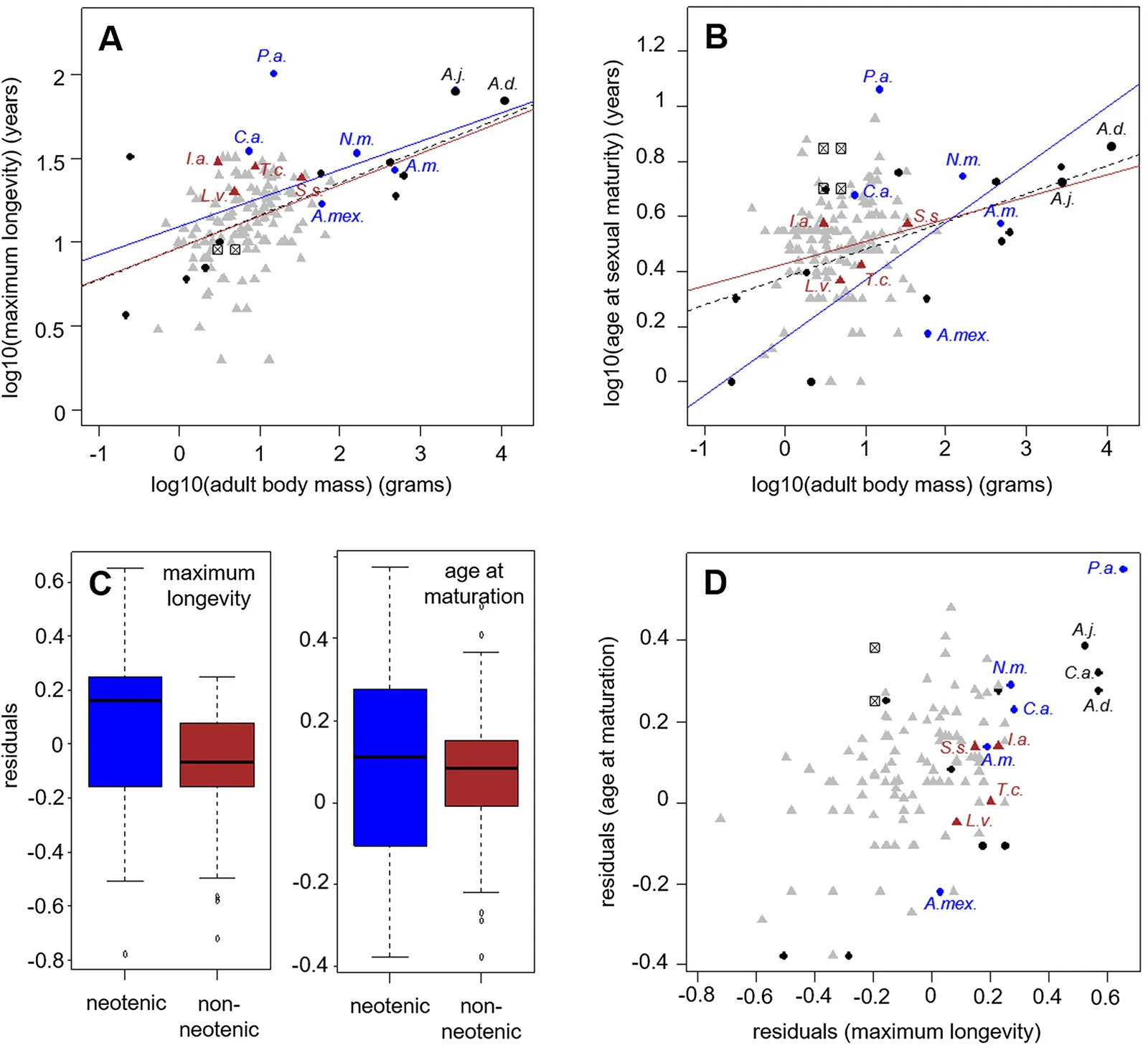
Results on differences in life history traits and strategies of obligate neotenic and non-obligate neotenic extant Caudata. Phylogeny-informed allometric regressions (PGLS) on maximum longevity (maxL), **A**, and age at maturation (ASM) **B**, respectively against adult body mass (BM). Non-obligate neotenic and obligate neotenic Caudata differ in their scaling of maximum longevity and age at maturation to adult body mass. PGLS models were calculated for all Caudata (dotted black line), non-obligate neotenic Caudata (brown line) and obligate neotenic Caudata (blue line) (Table 4A). **C**, obligate neotenic (blue) and non-obligate neotenic (brown) extant caudates show different patterns in the deviation of their maxL and ASM values from the allometric regression line across all caudates (residuals). After accounting for the allometric effect of adult body mass on the trait (residuals), obligate neotenic and non-obligate neotenic caudates do not differ in their mean maxL and ASM values, respectively (pANCOVAs, Table 4B). Obligate neotenic species show a significant larger dispersion of residuals than non-obligate neotenic species for both traits. **D**, the association in maxL and ASM residuals differs significantly between obligate neotenic and non-obligate neotenic species. Wilks’ λ indicates a weak separation of both groups (λ = 0.880). Symbols (A, B, D): circles = obligate neotenic caudates, triangles = non-obligate neotenic caudates, blue circles = the five modern obligate neotenic species studied herein, brown triangles = the four non-obligate neotenic modern species studied herein, crossed squares = *Apateon* spp. We assumed two values on BM for *Apateon* spp. (3 and 5 g), one on maxL and two on ASM [46–48]. Abbreviations of extant species (A, B, D): *C.a. = Calotriton asper*,* P.a. = Proteus anguinus*,* A. mex. = Ambystoma mexicanum*,* N.m. = Necturus maculosus*,* A.m. = Amphiuma means*,* I.a. = Ichthyosaura alpestris*,* L.v. = Lissotriton vulgaris*,* T.c. = Triturus cristatus*,* S.s. = Salamandra salamandra*,* A.j. = Andrias japonicus*,* A.d. = Andrias davidianus*,* C.a. = Cryptobranchus alleganiensis*. The osteohistology of *A. japonicus* and *A. davidianus* was subject to the study of Klein et al., [31]

The two traits, maximum longevity and age at maturation, increased significantly with an increasing adult body mass in all caudates, in obligate neotenic caudates and in non-obligate neotenic caudates (Fig. 4A, B, Table 4A). All slopes of the respective linear models were positive and differed significantly from zero and p-values indicated highly significant deviations (*p* < 10^− 16^) from zero for all slopes, except for maximum longevity in obligate neotenic species (*p* = 0.004) and age at maturation in non-obligate neotenic species (*p* = 0.012). Intercepts of all models were highly significant (*p* < 10^− 16^), except for that of the model on age at maturation in obligate neotenic species, for which the intercept was not significant. Phylogenetic signals assessed from Pagel’s λ values covered a broad range across caudates’ samples and traits (-0.326 to 1.007). Large overlaps in 95% confidence intervals of slopes and intercepts indicate no difference in the allometric models of obligate and non-obligate species for maximum longevity, whereas smaller and non-overlapping intervals in slopes indicate differences between both species samples for age at maturation (their intercept overlap) (Table 4A; Fig. 4).

**Table 4:** Results on differences in the allometric scaling of maximum longevity and age at maturation, respectively to adult body mass in different samples on extant Caudata

A)											
Sample	Trait	N	slope	p	95%CI(slope)	int	p	95%CI(int)	λ	95%CI(λ)	Res. s.e.
All	maxL	156	0.193	<10^-16^	(0.139, 0.248)	0.966	<10^-16^	(0.926, 1.001)	-0.023	(-0.023, 0.023)	0.238
Non- obligate neotenic	maxL	125	0.187	<10^-4^	(0.093, 0.280)	0.967	<10^-16^	(0.895, 1.039)	-0.025	(-0.025, -0.025)	0.223
Obligate neotenic	maxL	16	0.169	0.004	(0.064, 0.274)	1.094	<10^-16^	(0.952, 1.236)	-0.326	(-0.326, -0.326)	0.327
All	ASM	176	0.101	<10^-16^	(0.058, 0.144)	0.378	<10^-16^	(0.278, 0.479)	0.317	(0.043, 0.592)	0.187
Non-obligate neotenic	ASM	145	0.081	0.012	(0.018, 0.143)	0.428	<10^-16^	(0.352, 0.503)	0.122	(-0.067, 0.311)	0.162
Obligate neotenic	ASM	16	0.208	<10^-16^	(0.207, 0.209)	0.159	0.29	(-0.151, 0.470)	1.007	(1.007, 1.007)	0.309
**B**)											
Trait	df		F(slope)		p(slope)		F(int)	p(int)	F(mode)	p(mode)	
maxL	138		43.68		<0.001		5695.146	<0.001	0.051	0.821	
ASM	158		27.025		<0.001		111.348	<0.001	1.808	0.181	

The pANCOVAs indicated that mean values of maximum longevity and age at maturation that were corrected for adult body mass do not differ between obligate neotenic and non-obligate neotenic caudates (Table 4B). Thus, after accounting for the allometric effect of adult body mass on the respective trait, obligate neotenic and non-obligate neotenic caudates have similar average maximum longevities and ages at maturation, respectively. Obligate neotenic species, however, showed a significant larger dispersion of residuals than non-obligate neotenic species for both traits (Fig. 4C) as Ansari-Bradley tests clearly rejected variance homogeneity of trait residuals (Ansari-Bradley test; maximum longevity: AB = 326, *p* < 0.001; age at maturation: AB = 485.5, *p* < 0.001).

The Wilks’ λ test indicated significant, but small differences in the association of the residuals on maximum longevity and age at maturation between obligate neotenic and non-obligate neotenic species (Wilks’ λ = 0.880, χ^2^ = 14.988, df = 2, *p* < 0.001) (Fig. 4D).

## Discussion

### Microanatomy as reflected in medullary cavity size

For multi-individual samples, we observed a wide range in the sizes of the medullary cavities and thus bone compactness. This applies to *Apateon* spp., and humeri of *Ambystoma mexicanum* (but not to the femora of the latter), but not to *Triturus cristatus*. The female of the obligate neotenic *Necturus maculosus* and *Amphiuma means*, as well as the facultative neotenic *Calotriton asper* have tendentially the most compact bones (< 15%). All other taxa studied share a similar moderate size range in medullary cavities. The differences in the sizes of the medullary cavities between the male and female of *Necturus maculosus* might be either related to sex or ontogeny.

This overall high variability indicates that medullary cavity size is influenced by multiple and likely interacting factors such as individual variation, ontogenetic stage, presumably sex, environmental conditions [10, 28], and function.

Larger sample sizes per taxon, sex and ontogenetic stage, will be needed to disentangle these factors. The current results on observed sizes of medullary cavities of studied taxa neither indicate a correlation with phylogeny and lifestyle nor with developmental mode.

### Matrix

All adult taxa share an avascular lamellar matrix, except for *Necturus maculosus* that grew with parallel-fibred matrix. Parallel-fibred matrix is faster deposited than lamellar bone and indicates a higher growth rate in this species [6].

We here figure for the first time, cartilaginous matrix of early ontogenetic stages in petrographic ground sections (Suppl. Fig. S1A-D, 2 C, D, 3 C-F). The relatively large size of the *Proteus anguinus* individual (18 cm; Table 2) that still shows a pure cartilaginous midshaft is remarkable. However, this conforms to the observation made by Eggling (1911) [15], who described a cartilaginous rod surrounded by a bony shell for his “adult” *Proteus* specimens.

The identification of transforming osteoblasts scattered throughout the cortex of modern non-larval caudates was so far also unreported (Fig. 3). Although this feature is also documented in fully grown and old-aged individuals of *A. mexicanum*, in the other caudates it seems to be related to ontogeny, i.e. to not fully skeletal mature individuals. Besides the presence of transforming osteoblasts, the caudate samples show overall large but not very numerus osteocytes (Figs. 2 and 3; Suppl. info. SII; Suppl. Fig. S1-3).

We found no clear pattern in matrix related to the developmental mode of studied species. The observed osteoblasts might be related to slow or delayed mineralization processes and an overall prolonged development in caudates.

### Growth marks

We observed a difference in markedness of growth marks (distinct LAGs vs. faint alternating zones and annuli) in the petrographic ground sections. Distinct growth marks occur in taxa that live in seasonal environments (e.g., *Apateon* spp., *Amphiuma means*, *Necturus maculosus*, *Salamandra salamandra*, *Triturus cristatus*) but seems to be also related to an advanced ontogenetic stage (e.g., *Calotriton asper; Ichthyosaura alpetris*). *Ambystoma mexicanum* and *Proteus anguinus* lived in a rather stable aquatic environment of low seasonality in temperature and have a year-round activity phase and they show only faintly expressed growth marks. Besides a general presence of growth marks, we cannot reliably conclude a correlation between the markedness of growth marks and developmental mode. Environmental, ontogenetic and methodical issues (petrographic ground sections vs. stained microtomized sections) must be considered here as well.

### Differences in life history traits and strategies between extant obligate neotenic and non-obligate neotenic caudates

With our different statistical analyses, we aimed to find out whether life history traits and their associations differ between extant obligate neotenic Caudata and non-obligate neotenic Caudata. Statistically well-constrained differing allometric regressions on maximum longevity and age at maturation, respectively against adult body mass indicate the general pattern that these traits scale differently in obligate neotenic and non-obligate neotenic Caudata (Table 4; Fig. 4A, B). However, irrespective of whether modern species are obligate neotenic or not, similar-sized caudates show a large variability in their maximum longevities and ages at maturation (Fig. 4A, B). This suggests that these two traits cannot clearly separate similar-sized species of both groups from each other and that other factors must have a large influence on both traits. In extant Lissamphibia, adult body mass and thus other life history traits scaling to mass are strongly affected by environmental conditions [13, 14]. When accounting for the allometric effect of body mass on maximum longevity and age at maturation, obligate neotenic and non-obligate neotenic species do not differ in their mean values (Table 4; Fig. 4C) indicating no larger average trait values in the one than the other group. Conversely, obligate neotenic species scatter significantly stronger around the allometric regression line on all caudates than non-obligate neotenic species as variance homogeneity was clearly rejected for residuals on maximum longevity and age at maturation for both species’ traits (Fig. 4C). Particularly that neotenic taxa are rather variable in their life history traits, was also described by Lyons and Arbuckle [36]. Many obligate neotenic species including those that were subject to our osteohistological examination herein deviate considerably from the value predicted by the allometric regression model on maximum longevity and age at maturation, respectively calculated from all caudates (Fig. 4A, B). Likewise, a significant, but large Wilk’s λ value of 0.880 indicated that obligate neotenic and non-obligate neotenic species do slightly differ in their associations of maximum longevity and age at maturation when controlling the effect of adult body mass on both traits (residuals, Fig. 4D).

All these slight differences in trait and mass-corrected trait values found are not sufficient to clearly separate obligate neotenic from non-obligate neotenic species from each other as exemplified by several selected modern species including those studied herein (Fig. 4). Compared to all other caudates in our dataset, the obligate neotenic species *Andrias japonicus*, *Andrias davidianus*, *Cryptobranchus alleganiensis* (all Cryptobranchidae, [34]), and *Proteus anguinus* are best separated from all other caudates with respect to their association in the body mass corrected maximum longevities and ages at maturation (Fig. 4D). All these species live very long and mature late in their life. Particularly, *Proteus anguinus* shows a very high maximum longevity and late maturation for its comparatively small body mass (Fig. 4A, B). As the deviation of their maximum longevities and ages at maturation from the allometry on all caudates is small, their outlier position when plotting trait residuals against each other (Fig. 4D) is not informative on their development mode. For the facultative neotenic *I. alpestris* and the viviparous *S. salamandra*, ages at maturation are larger than predicted by the allometric regression on all Caudata and smaller than expected for their size in the facultative neotenic *L. vulgaris* and in *T. cristatus*, whereas maximum longevities of these four species are higher than expected from the respective line (Fig. 4A, B). This observation together with the observation that several obligate modern taxa show similar deviations from the average supports again that both life history traits are not informative on a taxon’s development mode (Fig. 4D).

In conclusion, the large variability observed in maximum longevities and ages at maturation and their different associations among each other and to adult body mass observed in obligate neotenic and non-obligate neotenic modern species (Fig. 4) preclude a use of these three traits to infer unequivocally whether a taxon is obligate neotenic or not.

The phylogenetic signal (λ values) calculated by the PGLS model on maximum longevity against adult body mass that was calculated for all caudates was small and not significant, whereas that on age at maturation was larger. These overall small λ values are corroborated by respective PGLS models calculated by Cejp and Griebeler [13] on modern Caudata and suggest that phylogeny did not constrain the change in maximum longevity in the evolutionary history of modern caudates and did weakly constrain the evolution in their ages at maturation. For the obligate neotenic caudates, the PGLS models on maximum longevity and age at maturation revealed contradicting results with respect to λ values. For maximum longevity, λ was moderately negative and significant and for age at maturation it was slightly larger than 1 and significant. This would suggest that in obligate neotenic Caudata phylogeny did not affect the evolution of maximum longevity, but rather strongly that of age at maturation. The low λ value on maximum longevity is consistent with that neoteny evolved independently multiple times in the evolutionary history of Caudata [2, 34]. The high value on age at maturation, however, contradicts the finding that age at maturation and maximum longevity correlate in modern caudates due to correlated evolution [13, 23]. We thus believe that this contradicting result on phylogenetic signals in obligate neotenic caudates resulted from our small sample sizes on this group (*N* = 16).

### *Apateon* spp.

The maximum longevity and age at maturation of *Apateon* spp. (adult body mass = 3 to 5 g, maximum longevity = 9 years, age at maturation = 5 to 7 years, Table 1) is rather similar to that of the similar-sized obligate neotenic dwarf waterdog *Necturus punctatus* (adult body mass = 4.2 g, maximum longevity = 10 years, age at maturation = 5 years, [13]), which is the smallest living species of Proteidae. This might provide further evidence that *Apateon* spp. had been neotenic. Several other similar-sized non-obligate neotenic caudates, however, show a similar association in adult body mass-corrected maximum longevities and ages at maturation seen in *Necturus punctatus* and *Apateon* spp. (Fig. 4D), which in turn questions that these deviations from the average clearly indicate obligate neoteny in *Apateon* spp.

Several points must be considered when assessing whether *Apateon* spp. was obligate neotenic or not from estimates on maximum longevity and age at maturation and their association (Fig. 4):

(1) Despite its overall morphological similarity to some modern neotenic caudates, *Apateon* spp. is not a lissamphib but a branchiosaurid temnospondyl taxon within Dissorophoidea, a clade considered by some authors as stem-group leading to Lissamphibia [33].

(2) *Apateon* spp. is among the first neotenic tetrapods documented in the fossil record, and as a possibly facultative neotenic animal, *Apateon* spp. might have had highly variable and/or differing life history traits at all.

(3) The maximum longevity (maximum observed age at death) and age at maturation of *Apateon* spp. is based on skeletochronological analysis and both age estimates could be incorrect for several reasons. Numbers of growth marks inferred either can under- or overestimate an individual’s actual age at death and maturation. Further on, indicators on its age at maturation might be misinterpreted. Despite problems in the interpretation and loss of growth marks, the number of preserved growth marks vary between elements of a single skeleton and anyway do not always correlate with actual age (e.g., [26, 32, 55, 59, 63]; this study our sample of axolotl). Contrary to the maximum longevities and ages at maturation analyzed in modern species, respective values on *Apateon* spp. are based on a small sample. Furthermore, our modern samples show how variable osteohistological signals in multiple individuals of the same taxon can be.

(4) Nothing is known about egg size, clutch size and breeding frequency in *Apateon* spp. In extant Lissamphibia, these traits are stronger affected by environmental conditions than by body mass [13]. These three traits as well as the three traits studied herein or any combination of them might be informative on the life history strategy of obligate neotenic and non-obligate neotenic taxa.

## Conclusions

We found no clear signal between medullary cavity size and developmental mode or lifestyle for our sample of modern caudates nor in *Apateon* spp. Problematic is that the variation in size of medullary cavities, which is the only proxy for microanatomy in the otherwise avascular matrix in our sample, increased with increasing sample size. This might point to a primary high intraspecific variability rather than to any ontogenetic, sex or environmentally related signal.

Matrix, and thus growth rate, is also not related to developmental mode or lifestyle. The occurrence of still transforming osteoblasts indicates ongoing bone mineralization processes and might indicate prolonged development but this is observed in all modern caudates included into our study and cannot be documented for *Apateon*. All samples show growth marks, but their markedness is largely related to environmental conditions and ontogenetic stage. Our sample clearly shows that not every individual necessarily reflects the population-level average but that intraspecific patterns are highly variable.

Due to a large interspecific variability in trait values across Caudata, allometric relations on maximum longevity and age at maturation against adult body mass and the association in species’ mass corrected maximum longevities and ages at maturation could not clearly separate obligate neotenic taxa from taxa that are not obligate neotenic. We found a tendency that these traits deviate more strongly from the values that are expected from the PGLS models on all Caudates in obligate neotenic than in non-obligate neotenic taxa. *Apateon* spp. shows such a substantial deviation from the allometric regression lines on maximum longevity and age at maturation in all Caudata, but similar-sized caudates that are not obligate neotenic show similar deviations which weakens our evidence for obligate neoteny in this extinct taxon. We also cannot rule out that inaccurate estimates of longevity and age at maturation that are based on skeletochronological data wrongly led to this finding.

Overall, a pattern related to osteohistology, microanatomy, or life history traits among the obligate neotenic and non-obligate neotenic extant caudate and extinct *Apateon* spp. was not identified. The high variability in all these features is most likely strongly linked to environmental conditions experienced by populations and taxa, underlining once more the high intraspecific variation and developmental plasticity in lissamphibians. Neoteny is an exceptional developmental pathway requiring flexible life-history traits, whereas osteohistology and microanatomy are likely to be more influenced by a certain chosen path.

## Supplementary Information

Below is the link to the electronic supplementary material.

Supplementary Material 1

## Data Availability

All data generated or analyzed during this study are included in this published article [and its supplementary information files]. The thin sections are available for first hand study in the respective institutes listed above.
