# Supplementary material for: Do osteohistology, microanatomy or life history traits show patterns linked to neotenic lifestyles in extant Caudata and the Permian temnospondyl Apateon?
Source: BMC Ecol Evol. 2026 Oct 1;26:90. doi: 10.1186/s12862-026-02577-x (PMC13654229; doi:10.1186/s12862-026-02577-x)
Supplement: Supplementary file 1 [file 12862_2026_2577_MOESM1_ESM.docx]

**Supplementary information**

**SI**

**The extinct branchiosaurid temnospondyl *Apateon* spp.**

*Apateon* is known from hundreds of specimens from the Early Permian of the Saar-Nahe Basin (Boy 1972). These abundant finds, include different ontogenetic stages, and thus morphology and development of *Apateon* is well documented (Schoch 2002; Fröbisch and Schoch 2009; Schoch and Fröbisch 2006). *Apateon* was a small axolotl-like animal with a body length of 5-12 cm. It retained external gills and other morphological “juvenile or larval” characters throughout its life, implying that it had been paedomorphic which was achieved through neoteny (i.e., delay in somatic development) (Schoch and Fröbisch 2006). Thus, it is hypothesized that *Apateon* reproduced without undergoing complete metamorphosis.

A terrestrial adult phase, i.e., a metamorphosed individual as an alternative developmental pathway is documented for one specimen of *A. gracilis* only (Schoch and Fröbisch 2006; Fröbisch and Schoch 2009). However, this implies that the possibility exists in *Apateon* spp. to metamorphose into a terrestrial form under particular conditions, although this seems to be rare.

*Apateon* had had a laterally flattened tail suggesting good swimming abilities, whereas the limb elements remained small and unlikely improve swimming capabilities. Maybe limbs were used to walk over the ground as the axolotl does (pers. obs. NK). *Apateon* lived in an ecosystem consisting of various lakes and ponds in a geographic region which is today called the Saar-Nahe Basin (SW Germany and E-France, Europe). These lakes were during the Early Permian levelled at a high altitude (intramountainous lakes), maybe about 2000m above sea level (Becq-Giraudon et al. 1996). The region was during the Early Permian within the tropical region; according to Boy and Sues (2000) approximately 10° north of the Equator. However, Richey et al. (2020) reported evidence of a double-peak glaciation maximum during the Asselian, with peaks in the early and late periods of this time span. The late glaciation peak of the Asselian falls into the time when *Apateon* spp. had lived and surely has affected all life in the lakes of the Saar-Nahe Basin. Glaciation means in general cooler global temperatures and widespread glacial activity, which even more effects climate in high altitude than low altitude environments. These lakes existed subsequently or in parts contemporaneously and were of different sizes and depths (Schindler 2007; Schoch 2025), likely also influencing growth, osteohistology, and life history traits of *Apateon*. It is unknown where and how *Apateon* exactly lived in these lakes. It is conceivable that it either hid in near shore vegetation or lived on the muddy ground at the bottom of the lakes. Feeding changed during ontogeny from filter feeding plankton to catching larger prey. Also, cannibalism is documented (Witzmann 2009). Modern lissamphibians such as the well-studied newt *Calotriton asper* show high plasticity in development, growth (i.e., life history) and behavior when living at different altitudes and habitats (lakes vs. streams) (e.g., amphibiaweb.org; Morrison and Hero 2003; Miaud and Guillaume 2005). *Apateon* spp. likely showed a similar plasticity (Sanchez et al. 2010 a, b).

Osteohistology and growth record of *Apateon* spp. was studied and described in detail by Sanchez et al. (2010a-c). They described avascular lamellar bone and numerous growth marks indicate a strong seasonal dependency of growth for *Apateon pedestris* and *Apateon caducus* from different localities. The maximum age in years was found to be 7 to 9 years for *Apateon* spp. with most specimens showing numerous non-annual growth marks, indicating strong seasonal dependency including an aestivation and hibernation period (Sanchez et al. 2010 a-c). Sexual maturity was found to start between 5 to 7 years, depending on locality.

**Life history traits and previous studies on osteohistology of the here studied modern neotenic Caudata.**

If not cited otherwise the information on the life history of modern lissamphibians was compiled from the website amphibiaweb.org and wikipedia.org.

***Ambystoma mexicanum***

*Ambystoma mexicanum* (axolotl), is a facultative neotenic caudate with a body size range of about 15–45 cm with most individuals attaining 23–28 cm. It retains external gills, has a finned swimming tail and four functional limbs, which are used to walk over the ground. It is endemic to the historic Lake Xochimilco system in the Valley of Mexico. This is a highly specialized habitat of canals, wetlands, and shallow lakebeds with cool, well-oxygenated, spring-fed lakes that historically offered stable environmental conditions and fairly constant temperatures (12°-22°C). Females lay several hundred eggs per clutch, attaching them individually or in small clusters to submerged vegetation or debris. Embryos develop over 10–14 days, hatchlings are about 10–12 mm and larvae begin active foraging almost immediately after hatching. During the species' first year of life, individuals grow rapidly, reaching up to 20 cm in total length. In their second year of life individuals can grow an additional 10 cm, after which the growth rate decreases (Zambrano et al. 2007). Individuals reach sexual maturity at approximately one year of age and at a body size of about 20 cm.

Although neotenic, neoteny is facultative in this species and metamorphosis can be induced experimentally through endocrine manipulation (Thyroxin/iodine) or is triggered by environmental stress (Chauvin 1876). However, such transitions are rare under natural conditions, why we treated it in our study as obligate neotenic. Population density influences growth, i.e., crowding suppresses growth and cannibalism occurs, the cannibalistic morphs then growing faster due to a higher protein content of food. *Ambystoma mexicanum* individuals retain a striking regenerative capacity throughout their lives (Vieira et al. 2019), making it an often-studied animal. However, its osteohistology and skeletochronology were not studied in detail before. Axolotl show rapid growth during their 1^st^ year of life, gathering 20 cm in total length in the wild; during 2nd year of life wild individuals can grow an additional 10 cm, then growth rate decreases.

*Ambystoma mexicanum* is well-studied in terms of its regeneration capacities (Bothe et al. 2021) but much less is known about its growth and skeletochronology. Riquelme-Guzmán et al. (2022) studied postembryonic development of the appendicular skeleton in *A. mexicanum* by using stained microtome sections and micro-CT data. One important finding of their study is that ossification of the appendicular skeleton starts when individuals reach a length of 10 cm. They further observed a continuous ossification in all skeletal elements of limbs in aged adult animals (5 to 20 years). *Ambystoma mexicanum* is under natural conditions always neotenic but metamorphosis can be induced experimentally through endocrine manipulation.

**Proteidae**

*Proteus anguinus* (olm) is a cave-dwelling salamander endemic to the Dinaric Karst region of Central and Southeastern Europe, including Slovenia, Croatia, Italy, and Bosnia-Herzegovina. It is the only species in its genus and exhibits a unique set of adaptations to life in complete darkness. The olm inhabits caves and karst systems with cold, stable, and nutrient-poor underground waters at relatively constant water temperatures around 10-12°C. The olm represents a remarkable example of evolutionary specialization, and it is highly sensitive to environmental changes and pollution. *Proteus anguinus* is obligatory neotenic, which is documented by the retainment of larval characteristics such as external gills throughout its life and that it never undergoes metamorphosis. The olm has an elongated, eel-like body, typically measuring between 20 and 30 centimeters, with small, reduced limbs. It has evolved an exceptional life history strategy with a predicted maximum lifespan of over 100 years (Voituron et al. 2011), and an adult average lifespan of 68.5 years. This longevity comes along with a highly energy-efficient lifestyle, in which they can remain motionless for extended periods, sometimes even years (one individual did not move for 7 years, average movements are 5m/year Balázs et al. 2019), conserving resources in the food-scarce cave environment.

Sexual maturity occurs at 15.6 years and females lay on average 35 eggs every 12.5 years (Balázs et al. 2019). Maximum clutch size is up to 70 eggs. Tadpoles are with 2 cm length relatively large when they hatch and live on yolk stored in the cells of the digestive tract for a month. Survival rates in the first few years are relatively low due to the extreme environmental conditions. The main reason for their low survival rates is food availability (i.e. starving due to lack of energy reserves). Predation pressure is also high for larvae by cave dwelling beetles, crustaceans, aquatic insects, worms, other olms (cannibalism!) and sometimes fishes’ prey on small olms. After having reached a certain size (i.e. established reserves), olms can survive for months if not years without eating. *Proteus anguinus* feeds primarily on small aquatic invertebrates, mollusks, and occasionally other small cave fauna including other olms. It is an ambush predator waiting motionless for its prey. Its opportunistic feeding strategy, extreme sensory adaptations, and energy-efficient metabolism allows it to survive in the nutrient-poor, dark environments of subterranean waters. The olm growth continuously slow throughout its life.

Eggeling (1911) described for an adult individual of *Proteus anguinus,* a cartilaginous rod surrounded by a bony shell, and the lack of a medullary cavity. The exceptional maximum longevity of 102 years assumed for *Proteus* *anguinus* with an average lifespan of 68.5 years, are the result of ecological studies (late sexual maturity, low reproduction rate, low mortality rates; low predatory pressure) on several populations (Voituron et al. 2011; Balázs et al. 2019) and are not based on skeletochronological data.

***Necturus* *maculosus***

*Necturus* *maculosus* (common mudpuppy) is a fully aquatic salamander, native to freshwater habitats in eastern North America. The taxon is a strong ecological indicator for clean and well‑oxygenated water in rivers, lakes, and streams. The species also needs suitable benthic substrates (rocks, logs, submerged debris) for shelter and foraging. It prefers temperatures about 10 to 20°C and avoids higher temperatures by hiding in deeper water. It hibernates on the bottom of water bodies. *Necturus* *maculosus* is obligatory neotenic, i.e., individuals remain aquatic throughout their life and retain larval-like features such as notably external gills. They have a robust and elongated body, two pairs of relatively short but well-developed limbs, and a laterally compressed tail, which mainly aids in aquatic locomotion. They move slowly along river or lake bottoms, or swim with eel- or fish-like undulations. Their diet is opportunistic and carnivorous: they feed on a wide variety of aquatic prey including insects, mollusks, crustaceans, worms and other invertebrates. Larger individuals may also take small fish or lissamphibian prey. They reproduce annually or biannually depending on species and environmental conditions. After egg-laying, the female often guards the nest until larvae hatch, which may take several weeks (roughly 5–7 weeks, depending on temperature and water conditions). Hatchlings emerge as fully aquatic larvae, already equipped with external gills and typical *Necturus* *maculosus* morphology. The larvae grow relatively fast during their 1^st^ years (Senning 1940). Sexual maturity occurs at around 4–6 years and at a body size of about 20 cm (depending on growth and environment) (Senning 1940). According to Senning (1940) lifespan is over 23 years whereas Cejp and Griebeler (2024) had 34yrs.

Senning (1940) did a thorough study of the histological growth record in *Necturus maculosus* as preserved in the parasphenoid, reporting and analyzing growth marks (i.e., incremental lines) in that element. He constructed a growth curve of 162 individuals, the oldest attaining an age of 23 years. He further noted that (Senning 1940:492) “the ages of most individuals can be determined with certainty to the fifteenth or sixteenth year. Beyond this age, due to slow growth, the incremental lines become too crowded to be counted accurately. It is probable that during senescence some years are not recorded.”

Senning (1940) further documented rapid growth until the 6/7-8year (8^th^ to 9^th^ growth season), then sexual maturity is reached and growth slows down markedly. The incremental lines are subject to individual variation but are widely spaced before maturity but increasingly

closer thereafter (Senning 1940). Body size-age correlation is not good during the first years of life. Buffrénil and Laurin (2021) described and figured a cross section of a petrographic ground section of a femur of *Necturus maculosus*. The avascular cortex is made of parallel-fibered tissue. The thin birefringent layer around the medullary cavity is interpreted by the authors as a Kastschenko’s line. Regularly deposited growth marks (likely representing zones and annuli) are obvious from the figure (Buffrénil and Laurin 2021:17.2B).

Skeletochronological analysis of the parasphenoid bones was applied to *Necturus maculosus* by Senning (1940). Buffrénil and Laurin (2021) described avascular parallel-fibered tissue in a petrographic ground section of a femur of *Necturus maculosus*. Regularly deposited growth marks are obvious from the figure of this section (Buffrénil et al. 2021a:17.2B).

***Amphiuma means***

The eel‑like salamander *Amphiuma* *means* (two-toed amphiuma) has a fully aquatic lifestyle, swimming via undulation. Its (vestigial) limbs and toes are strongly reduced. It is considered to be neotenic due to the retainment of gill‑slits in sexual mature individuals, although they have functional and efficient lungs. They inhabit slow waters (swamps, marshes, sluggish streams, drainage ditches, bayous and other wetlands) throughout much of the southeastern USA. *Amphiuma* *means* prefers temperature above 20°C and reduces its activity below 15°C. They hunt crayfish (a key prey item), aquatic invertebrates, small fish, frogs or smaller lissamphibians and have a biting and suction‑feeding behavior. During dry periods when their aquatic habitat dries up, *Amphiuma* *means* may burrow into moist mud or substrate and enter a dormant state (aestivation). Individuals have been found in burrows as deep as about one meter below the surface in dried‑up ponds or ditches. They are capable of surviving long periods without food, even for years in captivity. Depending on species and female size, they lay 50 to 200 eggs (max of ~350 eggs is recorded). Females guard the eggs which are laid on moisture land or nearshore, coiling around them during incubation, providing protection from predation, desiccation, and possibly maintaining humidity. Incubation takes four to five months, depending on environmental conditions. The larvae grow fast during the 1^st^ year of life, growing from ~5 cm up to 15 to 20 cm.

Bones of *Amphiuma* spp. had not been subject to osteohistological studies before.

***Calotriton asper***

The Pyrenean newt is endemic to the mountain ranges of the Pyrenees (Spain, France; Europe). It is adapted to life in cold, oxygen-rich, sometimes fast-flowing freshwater environments typical to mountain streams and lakes. In running waters, it lives under stones/rocks (Montori 1990). It occurs up to altitudes of 2500m NN. *Calotriton asper* shows variable life history traits depending on population and habitat type (stream vs lake, high or low altitude; Miaud and Guillaume 2005), including facultative neoteny in some mountain lake populations (53% of the adults retain gills) living in a stable aquatic environment (Oromi et al. 2014; Camarasa et al. 2020). Lacustrine populations, with facultative paedomorphosis, attained their maturity earlier (Camarasa et al. 2020). It is primarily carnivorous, feeding on aquatic invertebrates (insect larvae, aquatic arthropods (e.g., mayflies, stoneflies, dipterans). Egg development and time to metamorphosis depends on temperature and altitude (the colder/higher, the longer it takes). At a medium elevation (1000 m) larval stage lasts about 14 months and metamorphosis occurs in the 2nd summer. At higher elevations (2000 m) larval stage can last 2 yrs and metamorphose occurs in 3rd summer

(Boehme et al. 1999). At higher elevations the larvae can grow to 95 mm before metamorphosis.

Metamorphosis is in general slow and takes a month at 12ºC. Age structure varies between populations and sexes. Longevity is in average 20 years (Noellert and Noellert 1992)

Recorded are 26 years for a female and 20 years for a male (Montori 1990). Camarasa et al. (2020) mentioned a maximum age of 35 years. Growth rate and life history traits are highly dependent on habitat and altitude.

Skeletochronological studies on this species were conducted by several authors (Montori 1990; Oromi et al. 2014; Camarasa et al. 2020) using stained microtomized sections of wild populations. Growth marks are wider spaced in earlier ontogenetic stages when compared to older ontogenetic stages (Montori 1990; Buffrénil et al. 2021b). Femora show some endosteal resorption (Montori 1990). An additional interesting observation of Montori (1990) is that chromophily (i.e., the staining ability of LAGs) decreases after the 9^th^ to 11^th^ LAG ahead. Growth marks are very sharp and regular biannual growth cycles (double LAGs, split of LAGs), especially in mountain-dwelling populations have been recorded in *Calotriton* *asper* living at high altitude (Montori 1990). However, Oromi et al. (2014) did not observe double LAGs in their skeletochronological study on lake and stream-dwelling populations.

The matrix is described as parallel-fibred or finely lamellar periosteal bone tissue with a few simple vascular canals and rather obvious evidence of growth cycles (Francillon-Vieillot et al. 1990; Ricqlès et al. 1991). However, based on the figures published, large osteocyte lacunae are here misinterpreted as vascular canals, leaving the matrix of *Calotriton* *asper* avascular (pers. obs. NK, DKM).

Skeletochronology of *Calotriton asper* is well studied by using stained microtomized sections of different wild populations (Montori 1990; Oromi et al. 2014; Camarasa et al. 2020). For a more detailed summary of the results of the here mentioned studies please see Table 1 and the Supplementary information.

***Ichthyosaura alpestris***

*Ichthyosaura alpestris* (alpine newt) is a facultatively neotenic salamander (Sotiropoulos et al. 2007) with a body size range of 7–12 cm. Females are in general slightly larger than males.

It prefers forested land habitats in high altitude as well as in the lowlands. In the Alps it occurred up to 2,370 m. The species is restricted to central Europe. The range reaches from Denmark as a northern limit and runs southeastward to Romania and Bulgaria via the Carpathians and the Balkan Mountains. Towards the south of its range, most populations are found above 1,000 m. For reproduction it migrates to water bodies. Timing and duration of hibernation and reproduction depends on latitude and altitude. The clutch contains probably 73-190 eggs deposited singly or in short chains of 3-5 eggs over a few days.

The aquatic larvae hatch at 7–11 mm and grow up to 3-5 cm in around three months before metamorphosing. In the Ukrainian Carpathians, metamorphosis occurs in September, later than in other newt species. Larval hibernation and neoteny occurs frequently in this species. Some populations in the mountains consist mostly of neotenic individuals. The sexual maturity in normal individuals is reported for the age of two or three (males) to five (females) years.

At lower altitudes this occurs in males after around three years, and in females after four to five years. Lowland alpine newts can reach the age of ten. At higher altitudes, maturity is reached only after 9–11 years, and the newts can live for up to 30 years (Thiesmeier and Schulte 2010).

***Lissotriton vulgaris***

*Lissotriton vulgaris* (smooth newt) is a facultatively neotenic salamander (Litvinchuk 2001) with a body size range of 8–11 cm. Males are in general slightly larger than females. It prefers wood habitats but can be also found in field edges to parks and gardens. *Lissotriton vulgaris* spends most of the time on land and returns to water bodies only for reproduction. The largely nocturnal smooth newt occurs in the temperate belt of Europe and surroundings. Its abundance, decreases northwards and southwards, in high latitudes as well as in the dry steppe zone but generally tolerates/lives under a wide range of temperatures. Timing and duration of hibernation and reproduction depends on latitude and altitude.

The clutch consists of about 60-300 eggs, which are deposited singly or in small groups

Larvae hatch after 10 to 20 days. Larvae develop for around three months and metamorphosis is completed in the same year. However, in many cases larvae overwinter, in some cases they stay neotenic. They reach maturity after two to three years, and the adults live for up to a maximum of 14 years.

***Triturus cristatus***

For *Triturus cristatus* (northern crested newt) neoteny seems so far not documented because we found not any source mentioning neoteny for this taxon. It is a relatively large newt species. Males typically grow to a total length of 13.5 cm, while females can reach 16 cm. It is distributed through central Europe and is a forest-dweller which migrated to stagnant water bodies for reproduction. It is generally a lowland species but has been found up to 1,750 m in the Alps. Clutch size ranges between 70-600 (usually 150-200) eggs, which are deposited singly or in chains of 2-3 eggs.

Embryogenesis takes 12-20 days. The larval development is about 2.5-3 months or more. Metamorphosis occurs in late summer and autumn. Many larvae hibernate and complete their transformation in the next year. Juveniles take another year or two to reach maturity; this process can take longer in the north and at higher elevations. The larval and juvenile stages are the riskiest for the newts, whereas adults have a higher survival rate. Longevity is 7-9 years, although some individuals have lived for up to 17 years in the wild.

***Salamandra salamandra***

At 15–25 centimeters this salamander is one of Europe's largest salamanders^.^ Females are generally larger than males and possess relatively shorter extremities and tail.

*Salamandra salamandra* is widely distributed across southern and central Europe and prefers forests. They are most commonly found at altitudes between 250 meters and 1,000 meters, only rarely below (in Northern Germany sporadically down to 25 meters. However, in the Balkans or Spain they are commonly found in higher altitudes as well.

The fire salamander is typically a viviparous species and eggs develop internally.

Mostly they are ovoviparous (give birth to larvae) but some are viviparious (give birth to fully developed salamanders). Wolterstorff (1928) reported on one litter in which 24 normal larvae and 4 already fully developed salamanders were born. After a gestation period of 2-7 months, the female gives birth to 2-3 cm long larvae. The female deposits aquatic larvae or even fully developed young (i.e. fully formed metamorphs) into a body of water just as they hatch during its activity phase (February until autumn). Between 10 and 70 larvae have been counted per female with an average of 20 and 40 larvae. Up to 20 fully developed terrestrial juveniles can emerge from one female (Beukema et al. 2016). Larvae develop within 3-5 months depending on temperature and leave the water body as 4-7 cm long juveniles. In cave waters, development can take up to a year. They often overwinter and finish their metamorphosis in the next year. Contrary to egg-hatched larvae of other species, fire salamander larvae start active feeding just after birth. At 5-6 years, the fire salamanders reach sexual maturity: males are then of over 15 cm long and females over 16 cm (Seifert 1991). Longevity is 20-50 years (24 years according to AnAge Database of Animal Ageing; > 50 years in terrarium according to Boehme 1979). Neoteny is possible in fire salamander but seems to represent an atypical developmental mode or a prolonged time until metamorphosis is completed (Voitel 2007; <https://amphibienschutz.org/feuersalamander/>). Breeding has not been observed in neotenic fire salamanders.

**SII**

**Detailed osteohistological description in petrographic ground sections of modern taxa**

***Ambystoma mexicanum***

The humeral midshaft section of the larva (BIOP-PAL-186; Table 2) showed a large free medullary cavity (~60% of the cross-sectional diameter) filled by a black substance likely representing organic material on which’s’ margin “clastic” cells are visible (Fig. S1A). The medullary cavity is surrounded by a faint translucent avascular matrix, except for some erosional cavities. This matrix shows no optical features comparable to any bony matrix or calcified cartilage. The matrix contains one large oval cell and a few scattered smaller black roundish cells. We interpret the large cell as a hypertrophic chondrocyte and the other cells as transitional forms of osteoblasts developing into osteocytes (Franz-Odendaal et al. 2006); the matrix is thus cartilage.

The humeral midshaft section of the medium sized specimen ZFMK 83269 has an even larger medullary cavity (~80% of the cross-sectional diameter) (Fig. S1C, D). The avascular cortical matrix is highly organized lamellar bone but with a low degree of mineralization because collagen fibers are only poorly visible under cross polarized light. The matrix contains a mixture of scattered cells which are round to spindle-shaped (Fig. S1Q). Some are typical osteocyte lacunae surrounded by canaliculi; others are round-oval and smooth showing distinct nuclei and no canaliculi (Fig. S1Q), interpreted as osteoblast being in transitional stages towards osteocyte lacunae (Franz-Odendaal et al. 2006). This matrix represents a transitional stage between cartilage and mineralized bone, indicating ongoing mineralization of collagen fibers. The matrix is divided into four layers, interpreted as growth marks, as indicated by faint changes in color (Fig. S1Q).

The size of the humeral medullary cavities of the individuals from the larger and ontogenetic older specimens is in BIOP-PAL-175 60%, in BIOP-PAL-176 30%, and in BIOP-PAL-177 80% of the cross-sectional diameter (Table 2). All midshaft femoral sections (BIOP-PAL-175; BIOP-PAL-176; BIOP-PAL-177) have a cross-sectional diameter of about 65%. The humeral and femoral matrices of BIOP-PAL-175 and BIOP-PAL-176 are well mineralized, nicely exposing collagen fiber orientation of lamellar bone (Fig. S1E-L). However, BIOP-PAL-175 also shows a mixture of osteocyte lacunae and mature or transforming osteoblasts in its outer cortices, indicating ongoing mineralization of the cortex (i.e., the osteoid; Fig. S1R). The avascular matrices of all specimens are scattered by large osteocyte lacunae surrounded by canaliculi, which are however not very numerous. Cyclicity is visible although it is difficult to discern distinct rest lines from the high (lamellar) organization of the matrix. Several growth cycles are distinguishable by rest lines, likely representing lines of arrested growth (LAGs) and/or by changes in color and organization (lower and higher organized lamellar bone). However, the number of counted growth marks are always well below the actual age of these individuals (Table 2). The elements with a large medullary cavity show an even less complete growth record.

With respect to visibility of collagen fibers under cross polarized light, the cortical matrix of the humerus of BIOP-PAL-177 is less mineralized compared to that of BIOP-PAL-175 and BIOP-PAL-176 (Fig. S1R-T). The matrix of BIOP-PAL-177 is similar to the low mineralized matrix visible in ZFMK 83269. The humerus of BIOP-PAL-177 also shows less distinct collagen fibers than the femur of the same individual. Because none of these two elements were ever bitten off (pers. obs. NK), they both represent original bones and are not regenerated. Humerus BIOP-PAL-176 depicts in ventral direction few scattered erosion cavities (Fig. S1I, J).

Humerus and femur (BIOP-PAL-177) each have a lining of their medullary cavities with a very thin layer of endosteal bone that does not contain any osteocyte lacunae. Both elements of BIOP-PAL-175 have a distinct lining of their medullary cavities by endosteal bone containing osteocyte lacunae. The humeral section of BIOP-PAL-175 shows a Kastschenko’s line. Humerus BIOP-PAL-176 has an exceptional thick inner layer of lamellar bone that contains numerous osteocyte lacunae. This thick layer is surrounded by a much thinner and brighter layer of endosteal lamellar bone, which has no osteocyte lacunae (Fig. S1J).

***Necturus* *maculosus***

The midshaft cross section of the female (ZFMK 83281) is round with a small medullary cavity (12% of the cross-sectional diameter), whereas that of the male (ZFMK 83280) is more elliptical with a larger medullary cavity (36% of the cross-sectional diameter) (Table 2; Fig. S2I-L). The differences in shapes of cross sections and sizes of medullary cavities are confirmed by micro-CT pictures taken along the shaft of both elements and are not related to differences in sampling location. The inner margin of the humeral medullary cavity of the female is lined by a thin layer of endosteal bone containing no osteocyte lacunae. The endosteal bone in the male section (ZFMK 83280) is lined by cartilage, representing a Kastschenko’s line. Both sections are avascular and show no cortical erosion. The matrix is in the male section highly organized parallel-fibered matrix, relatively regularly scattered with large osteocyte lacunae, which are more numerous at the medial side where the cortex is thickest. In the section of the female the matrix is lamellar bone. In both samples, the innermost third of the matrix appears more organized than the surrounding matrix. The male section shows some maturing osteoblasts between the osteocyte lacunae. The cortex is in both sections well stratified by alternating zones and annuli ending in LAGs (Table 2). Both samples have distinct Sharpey’s’ fibers around the cross section extending from the inner to the outer cortex.

***Proteus anguinus***

The three humeral cross sections are round and all have a small medullary cavity (Table 2). The medullary cavities of the two larger specimens (ZFMK 860071, ZFMK 86001) are each lined by a thin layer of endosteal bone showing no osteocyte lacunae (Fig. S2E-H). The smallest specimen (ZFMK 86031) shows an avascular matrix like that of the axolotl larva (BIOP-V 2025-I) with no optical features comparable to any bony matrix or calcified cartilage (Fig. S2C, D) and is interpreted as cartilage as well. It, however, does not contain any cells. Radial structures (holes) reaching from the inner to the outer cortex along the posterior bone side are interpreted as Sharpey’s fibers (Fig. S2C, D). Sharpey’s fibers leaving similar traces in the cortex were also described for the giant salamander *Andrias japonicus* (Klein et al. 2024). The avascular cortex of the two larger specimens is made of lamellar matrix. Both cortices show numerous Sharpey’s fibers (Fig. S2F, H). In the medium sized specimen (ZFMK 86071), large, scattered osteocyte lacunae occur except for the dorsal side which has no osteocyte lacunae (Fig. S2G, H). The innermost third of the cortex of ZFMK 86001 is made by a thick layer of highly organized lamellar bone that shows no osteocyte lacunae (Fig. S2E, F). This area is divided into two layers by color and likely represents a phase of growth during an early ontogenetic stage. A similar inner ring is also visible in ZFMK 86071, but it is less pronounced. ZFMK 86031 shows an inner area which is white compared to the light brown rest of the cartilaginous matrix. However, no difference in the organization of the cartilaginous matrix is visible. Growth marks in form of alternating matrix organization representing zones and annuli, are visible in both larger specimens (Table 2; Fig. S2E-H). The cortex of ZFMK 86001 and ZFMK 86031 are both surrounded by soft tissue. In ZFMK 86031 a thick layer of soft tissue is attached to the posterior bone side. This is also the direction to which the Sharpey’s fibers run. In ZFMK 86001 the soft tissue consists of two layers which are tightly attached to the bone. The innermost layer might represent a ring of not fully mineralized matrix (periosteum), whereas the outer layer might represent soft tissue like muscles and/or skin.

***Amphiuma* *means***

The femoral cross section at midshaft is round with a small medullary cavity (14% of the cross-sectional diameter), which is surrounded by a thin layer of endosteal bone. The matrix is avascular and consists of lamellar bone, scattered with few large osteocyte lacunae (Fig. S2M, N). Growth marks are clearly visible in form of alternating zones and annuli (Fig. S2N).

***Calotriton asper***

The petrographic ground section of a metamorphosed individual (ZFMK 39157) shows an

small (10%) oval medullary cavity and avascular lamellar matrix with large osteocyte lacunae intermixed with mature and transforming osteoblasts (cells with nuclei and no canaliculi) (Franz-Odendaal et al. 2006) (Fig. S2A, B). The osteocyte lacunae and osteoblasts are much rarer at the dorsal bone side. The medullary cavity is surrounded by an inner ring of higher organized lamellar tissue containing some mature osteoblasts. The matrix shows stratification by growth marks which are however difficult to differentiate from the highly organized lamellar bone (Table 2).

***Ichthyosaura alpestris***

The size of the round oval medullary cavity in the petrographic ground section of individual BIOP-PAL-180 is in the humeral section 40% and in the femoral section 37.5%. The matrix is avascular lamellar matrix (Fig. S3H, I). Both elements show a thick layer of lamellar bone in their innermost cortex. Both elements show 2 to 3 alternating zones and annuli which are not very pronounced. Although the matrix does not show transforming osteoblasts, the degree of mineralization seems to be low in both sections.

***Triturus cristatus***

The size of the medullary cavity of BIOP-PAL-182a is in the humeral section 35.8% and in the femoral section 58.4% (Fig. S3A, B), it is not measurable in the humerus of BIOP-PAL-182b and 65% in its femur (Fig. S3 C, D), and 41.6% and 64% in BIOP-PAL-182c (Fig. S3 E, F). The matrix is avascular lamellar bone in the fully grown specimen (BIOP-PAL-182a). The cortex still contains few transforming osteoblasts. This individual shows 3 growth cycles (2LAGs) in its humerus. In the femur the growth marks are only faint. Both smaller specimens consist of cartilage or a very low mineralized bone. In any case mineralization seems to have started as indicated by the presence of osteoblasts.

*Salamandra salamandra*

The size of the medullary cavity of BIOP-PAL-178 is in the humeral section 26.6% and in the femoral section 38.7% (Fig. S3G, J-L). The matrix is avascular lamellar bone. The cortex of both sections shows few transforming osteoblasts. Growth marks are well visible in both sections. We count 6-7 alternating zones and annuli each ending in a LAG in the humerus. the femur shows one growth mark less.

**References**

AmphibiaWeb. (2026). Available at: https://amphibiaweb.org/ [Accessed January 2026].

Balázs G, Lewarne B, Herczeg G. Extreme site fidelity of the olm (*Proteus anguinus*) revealed by a long-term capture–mark–recapture study. J. Zool 2019;311(2):99-105

Becq-Giraudon JF, Montenant C, Van den Driessche J. Hercynian high-altitude phenomenon the French Massif Centrale: tectonic implications. Paleogeogr. Paleoclimatol. Paleoecol. 1996;122:227-41.

Beukema W, Speybroeck J, Velo-Antón G. Salamandra. Curr Biol. 2016;26(15):R696-R697.

Boehme, W. 1979: Zum Höchstalter des Feuersalamanders *Salamandra salamandra* (L.): Ein wiederentdecktes Dokument aus der Frühzeit der Terraristik (Amphibia: Caudata: Salamandridae). Salamandra 15(3): 176-179.

Boehme W, Grossenbacher K, Thiesmeier B. Handbuch der Reptilien und Amphibien Europas. Band 4/I: Schwanzlurche (Urodela)*.*Aula-Verlag, Wiesbaden: 1999.

Boy JA. Die Branchiosaurier (Amphibia) des saarpfälzischen Rotliegenden (Perm, SW-Deutschland). Abh. Hess. Landesamtes Bodenforsch. 1972;65:1-137.

Boy JA, Sues HD. Branchiosaurs: larvae, metamorphosis and heterochrony in temnospondyls and seymouriamorphs. In: Heatwole H, Carroll RL, editors. Amphibian Biology 4. Palaeontology, Chipping Norton, NSW, Australia: Surrey Beatty and Sons. 2000. p. 1150-97.

Bothe V, Mahlow K, Fröbisch NB. A histological study of normal and pathological limb regeneration in the Mexican axolotl *Ambystoma mexicanum*. J. Exp. Zool. B: Molecular and Developmental Evolution. 2021;336(2):116-28.

Buffrénil V de, Laurin M. Lissamphibia. In: Buffrénil de V, Zylberberg L, Ricqlès de A, Padian K, editors. (eds). Vertebrate Skeletal Histology and Paleohistology. Boca Raton, Florida: CRC Press. 2021. p. 345-59.

Buffrénil V de, Quilhac A, Castanet J. Cyclical growth and skeletochronology. In: Buffrénil de V, Zylberberg L, Ricqlès de A, Padian K, editors. Vertebrate Skeletal Histology and Paleohistology. Boca Raton, Florida: CRC Press. 2021. pp 626-40.

Camarasa S, Oromi N, Sanuy D, Amat F. Demographic Traits Variation in a Pyrenean Newt (*Calotriton asper*) among Lacustrine and Stream Populations. Diversity. 2020;12:471.

Chauvin M. v. Ueber die Verwandlung der mexicanischen Axolotl in *Ambystoma*. Z. Wiss. Zool. 1876;27:522-35.

Cejp B, Griebeler EM. Body Mass Shapes Most Life History Traits and a Fast-Slow Continuum in Amphibians. Ecol. Evol. 2024;14:10.

Eggeling H. Der Aufbau der Skeletteile in den freien Gliedmassen der Wirbeltiere; Untersuchungen an urodelen Amphibien. Jena: Fischer. 1911. p.1-324.

Francillon-Vieillot H, Buffrénil V de, Castanet J, Géraudie J, Meunier FJ, Sire JY, Zylberberg L, Ricqlès d. A. Microstructure and mineralization of vertebrate skeletal tissues. Skeletal Biomineralization: Patterns, Processes and Evolutionary Trends 1990;1:471-530.

Franz-Odendaal TA, Hall BK, Witten PE. Buried alive: how osteoblasts become osteocytes. Dev. Dyn. 2006;235(1):176-90.

Fröbisch NB, Schoch RR. The largest specimen of *Apateon* and the life history pathway of neoteny in the Paleozoic temnospondyl family Branchiosauridae. Foss. Rec. 2009;12:83-90

Hagstrôm T. Growth studies and ageing methods for adult Triturus vulgaris L. and *Triturus cristatus* Laurenti (Urodela, Salamandridae). Zool. Scripta. 1977;6:61-68.

Klein N, Konietzko-Meier D, Kalita S, Noda M, Ishikawa S, Taguchi Y, Anzai A, Hayashi S. Unique bone histology of modern giant salamanders: a study on humeri and femora of *Andrias* spp. Zool. Let. 2024;10:18.

Litvinchuk S. First record of paedomorphosis for the smooth newt (*Triturus vulgaris*) from Ukraine. Russian Journal of Herpetology. 2001,8:7.

Miaud C, Guillaume O. Variation in age, body size and growth among surface and cave-dwelling populations of the Pyrenean newt, *Euproctus asper* (Amphibia; Urodela). Herpetol. 2005;61(3):241-49.

Montori, A. Skeletochronological results in the Pyrenean newt *Euproctus asper* (Duge` s, 1852) from one Pyrenean population. Ann. Sci. Nat., Zool. (Paris). 1990;11:209-211.

Morrison C, Hero JM. Geographic variation in life-history characteristics of

amphibians: a review. J. Anim. Ecol. 2003;72:270-79.

Noellert A, Noellert C. Die Amphibien Europas. Stuttgart, Germany: Franckh-Kosmos Verlags-GmbH and Company. 1992.

Oromi N, Amat F, Sanuy D, Carranza S. Life history trait differences between a lake and a stream-dwelling population of the Pyrenean brook newt (*Calotriton asper*). Amphibia-Reptilia 2014;35(1):53-62.

Richey JD, Montañez IP, Goddéris Y, Looy CV, Griffis NP, DiMichele WA. Influence of temporally varying eatherability on CO2-climate coupling and ecosystem change in the late Paleozoic. Clim. Past 2020;16:1759-75.

Ricqlès Ade. Curtis TJW, Currey JD. et al. Comparative microstructure of bone. Bone 1991;3:1-78.

Riquelme-Guzm C, Schuez M, Böhm A, et al. Postembryonic development and aging of the appendicular skeleton in *Ambystoma mexicanum*. Devel. Dyn. 2022;251(6):1015-1034.

Sanchez S, Ricqlès de A, Schoch RR, Steyer JS. Developmental plasticity of limb bone microstructural organization in *Apateon*: histological evidence of paedomorphic conditions in branchiosaurs. Evol Dev. 2010;12(3):315-28.

Sanchez S, Germain D, Ricqlès de A., Abourachid AA, Goussard F, Tafforeou P.

Limb-bone histology of temnospondyls: implications for understanding the diversification of palaeoecologies and patterns of locomotion of Permo-Triassic tetrapods. J. Evol. Biol. 2010;23:2076-90.

Sanchez S, Steyer S, Schoch RR, Ricqlès de A. Palaeoecological and palaeoenvironmental influences revealed by long-bone palaeohistology: the example of the Permian branchiosaurid *Apateon*. Geol. Soc. Lond., Spec. Publ. 2010;339:139-49.

Seifert D. 1991. Untersuchungen an einer ostthüringischen Population des Feuersalamanders (*Salamandra salamandra*).Artenschutzreport, 1:1-16.

Senning WC. Study of age determination and growth of *Necturus maculosus* based on the parasphenoid bone. Am. J. Anat. 1940;66(3):483-95.

Schindler T. Geologie, Stratigraphie und Genese des permokarbonischen Saar-Nahe-

Beckens. In: Schindler T, Heidtke UHJ, Editors. Kohlesümpfe, Seen und Halbwüsten.

Dokumente einer 300 Millionen Jahre alten Lebewelt zwischen Saarbrucken und Mainz; Bad Dürkheim, Germany: Pollichia-Sonderveröffentlichung 2007;10:4-37.

Schoch RR. The evolution of metamorphosis in temnospondyls. Lethaia 2002;35:309-27.

Schoch RR. Morphological disparity, stratigraphic distribution and evolutionary history of the temnospondyl *Sclerocephalus* from the late Palaeozoic of Germany. Palaeodiv. 2025;18:151-95.

Schoch RR, Fröbisch NB. Metamorphosis and Neoteny: Alternative pathways in an extinct Amphibian clade. Evol. 2006;60(7):1467-75.

Sotiropoulos K, Eleftherakos K, Džukić G, Kalezić ML, Legakis A, Polymeni RM Phylogeny and biogeography of the alpine newt *Mesotriton alpestris* (Salamandridae, Caudata), inferred from mtDNA sequences. Mol. Phylogenet. Evol. 2007,45:211-226.

Thiesmeier, B.; Schulte, U. (2010). Der Bergmolch: im Flachland wie im Hochgebirge zu Hause [The alpine newt: at home in the lowlands and the high mountains]. Beihefte der Zeitschrift für Feldherpetologie (in German). Vol. 13. Bielefeld, Germany: Laurenti-Verlag.

Vieira WA, Wells KM, McCusker CD. Advancements to the Axolotl Model for Regeneration and Aging. Gerontology. 2020;66(3):212-222.

Voituron Y, Fraipont de M, Issartel J, Guillaume O, Clobert J. Extreme lifespan of the human fish (*Proteus anguinus*): a challenge for ageing mechanisms. Biol. Let. 2011;7(1):105-7.

Witzmann F. Cannibalism in a small growth stage of the Early Permian branchiosaurid *Apateon gracilis* (Credner, 1881) from Saxony. Foss. Rec. 2009;12:7-11.

Wolterstorff W. 1928: Vollmolch-gebärende Feuersalamander aus Oviedo**.** *Bl.* Aquar. Terrar. Kde. 39:132-13.

Zambrano L, Vega E, Herrera MLG, Prado E, Reynoso V.H. A population matrix model and population viability analysis to predict the fate of endangered species in highly managed water systems. Anim. Conserv. 2007;10(3):297-303.

**Supplementary figures**

**Figure S1. Osteohistology and microanatomy in petrographic ground sections in *Ambystoma mexicanum*.** For further details on size and other information of specimens see Table 2 and SII. Humeral cross section of larva (BIOP-PAL-186) under plane polarized **A**, and cross polarized light **B**. Humeral cross section of ZFMK 83269 under plane polarized **C**, and cross polarized light **D**. Humeral cross section of BIOP-PAL-175 under plane polarized **E**, and cross polarized light **F**. Femoral cross section of BIOP-PAL-175 under plane polarized **G**, and cross polarized light **H**. Humeral cross section of BIOP-PAL-176 under plane polarized **I**, and cross polarized light **J**. Femoral cross section of BIOP-PAL-176 under plane polarized **K**, and cross polarized light **L**. Humeral cross section of BIOP-PAL-177 under plane polarized **M**, and cross polarized light **N**. Femoral cross section of BIOP-PAL-177 under plane polarized **O**, and cross polarized light **P**. **Q,** Cortical detail of ZFMK 83269 showing osteocyte lacunae and mature and transforming osteoblasts as well as growth layers under plane polarized light. **R,** Cortical details of humerus BIOP-PAL-176 showing the osteoid with osteoblasts in the outer cortex. Collagen fibers are visible under cross polarized light. **S-T**, Cortical details of humerus BIOP-PAL-177 under cross-polarized light with no discernable collagen fibers in the matrix.

**Figure S2. Osteohistological and microanatomical details in petrographic ground sections of neotenic caudates.** For further details on size and other information of specimens see Table 2 and SII. **A-N**, Modern neotenic Caudata. **O-Q**, *Apateon* spp.

Humeral cross section of *Calotriton asper* (ZFMK 39157) under plane polarized **A**, and cross polarized light **B**. **C-H** Humeral cross sections of *Proteus anguinus*. ZFMK 86031 under plane polarized **C**, and cross polarized light **D**. ZFMK 86071 under plane polarized **E**, and cross polarized light **F**. ZFMK 86001 under plane polarized **G**, and cross polarized light **H**. **I-L**, Humeral cross sections of *Necturus maculosus.* Female ZFMK 83281 under plane polarized **I**, and cross polarized light **J**. Male ZFMK 83280 under plane polarized **K**, and cross polarized light **L**. **M-N**, Humeral cross section of *Amphiuma means* (ZFMK 32181) under plane polarized light **M**, and cross polarized light **N**. **O-Q,** Humeral cross sections of *Apateon* spp. all under plane polarized light. **O**, *Apateon pedestris* (SMNS 55018) from Niederkirchen **P**, *Apateon caducus* (GPIM 1572) from Erdesbach, and **Q**, *Apateon pedestris* (SMNS 54981) from Odernheim.

**Figure S3. Osteohistological and microanatomical details in petrographic ground sections of neotenic caudates.** For further details on size and other information of specimens see Table 2 and SII. **A-F**, Humeri and femora of *Triturus cristatus* of different ontogenetic stage under cross polarized light; **A**, humeral and **B**, femoral cross section of BIOP-PAL-182a, a large mature specimen. **C,** humeral and **D**, femoral cross section of medium sized specimen BIOP-PAL-182b. **E,** femoral and **F,** humeral cross section of small specimen BIOP-PAL-182c. **G**, humeral cross section of *Salamandra salamandra* under plane polarized light. **H**, humeral and **I**, femoral cross section of *Ichthyosaura alpestris* under plane polarized light. **J**, humeral cross section of *Salamandra salamandra* under cross polarized light. K, humeral and L, femoral cross section of *Salamandra salamandra* under plane and cross polarized light.
